# Modernizing Livestock Operations: Smart Feedlot Technologies and Their Impact

**DOI:** 10.3390/ani16081244

**Published:** 2026-04-18

**Authors:** Son D. Dao, Amirali Khodadadian Gostar, Ruwan Tennakoon, Wei Qin Chuah, Alireza Bab-Hadiashar

**Affiliations:** 1 School of Engineering, RMIT University, Melbourne, VIC 3000, Australia; son.dao@rmit.edu.au (S.D.D.); amirali.khodadadian@rmit.edu.au (A.K.G.); wei.qin.chuah@rmit.edu.au (W.Q.C.); 2 School of Computing Technologies, RMIT University, Melbourne, VIC 3000, Australia; ruwan.tennakoon@rmit.edu.au

**Keywords:** precision livestock farming, beef cattle, feedlot, computer vision, animal welfare, smart agriculture

## Abstract

Smart feedlots are increasingly adopting Precision Livestock Farming technologies to enable continuous, individual-animal monitoring and more proactive management in intensive beef production systems. This narrative review synthesises evidence from approximately 350 academic publications, of which 117 are formally cited, complemented by industry deployments and the authors’ experience in smart feedlot system development. We cover enabling digital infrastructure (power, sensing networks, wireless connectivity, and gateways), animal identification and sensing (RFID, automated weighing, wearables, and pen-side sensors), machine vision (RGB, thermal, and multispectral imaging from fixed and mobile platforms), and AI-based analytics and decision support for health, welfare, performance, and environmental management. Across the literature, key components have progressed beyond proof-of-concept toward operation under commercial constraints. Reported outcomes include reduced reliance on routine pen-rider observation and yard handling, earlier triage of emerging morbidity risk and behavioural change, and more standardised welfare auditing. Vision-based methods are repeatedly validated against trained human scorers in both on-farm and abattoir contexts, while automated weighing and image-based liveweight estimation support higher-frequency growth monitoring with low single-digit percentage error in representative studies. Precision feeding and targeted supplementation are associated with improved feed utilisation and reduced resource wastage, although effectiveness and adoption vary across animal classes and production stages. We identify priorities for robust, scalable deployment: resilient communications in harsh environments, appropriate edge–cloud partitioning under intermittent connectivity, and interoperable multi-sensor data fusion to deliver trustworthy alerts and actionable insights. Persistent barriers remain cost, durability, maintenance burden, integration and interoperability, data governance, and workforce capability.

## Simple Summary

Beef cattle feedlots are large, intensive operations where managing the health, welfare, and growth of thousands of individual animals is a significant challenge. Traditional approaches rely heavily on manual observation, which is labour-intensive, intermittent, and often unable to detect early signs of illness or poor welfare. This review examines how a range of digital technologies, including cameras, sensors, radio-frequency identification tags, automated weighing systems, and artificial intelligence, are being used to monitor individual animals continuously and non-invasively in modern feedlots. We summarise evidence from approximately 350 published studies and industry reports, of which 117 are formally cited, covering technologies for animal identification, health and behaviour monitoring, feeding and water intake tracking, and data-driven decision support. We find that several of these technologies have progressed beyond laboratory testing toward practical use in commercial settings, with reported benefits including earlier detection of sick animals, reduced labour requirements and more consistent welfare assessment. However, significant challenges remain, including the high cost of equipment, the difficulty of operating sensitive technologies in harsh outdoor environments, limited connectivity in rural areas, and the need for better integration between different systems. We identify priority areas for future research and highlight the growing potential of artificial intelligence tools, including large language models, to support more autonomous and responsive feedlot management in the future.

## 1. Introduction

### 1.1. Background and Motivation

The global beef market, valued at over USD 450 billion annually (e.g., USD 459.9 bn in 2024 according to Research and Markets [1]), is experiencing sustained growth in demand, particularly in emerging economies. Producers face increasing pressure from rising input costs and heightened regulatory and consumer expectations around animal welfare, sustainability, and supply chain transparency [2]. In this context, the adoption of digital monitoring and automation technologies in intensive livestock systems has been proposed as a means of improving both operational efficiency and welfare outcomes [3]. Effective cattle health monitoring in feedlots encompasses early disease detection, behavioural tracking, precision feeding, and real-time decision support. Each of these has been identified as critical for enhancing productivity and animal welfare outcomes. Earlier identification of at-risk animals and more timely, targeted interventions have been associated with reduced mortality, improved feed efficiency, and enhanced sustainability in feedlot management. Traditional manual health monitoring in feedlots is labour-intensive, intermittent, and prone to missing early signs of disease or distress, with treatment costs reported at up to $15–20 per animal animal, excluding performance losses [4,5]. The absence of continuous individual-animal data limits producers’ ability to intervene early, often resulting in elevated mortality rates and increased operational costs. For example, automated cattle health surveillance has been reported to reduce disease-related interventions by 15% and cut labour costs by over 10,000 annually in a 500-head Texas feedlot, while computer vision-based systems have demonstrated cost savings of approximately 420 per cow per year through improvements in lameness detection and body condition monitoring [6,7]. The automation of feedlot animal welfare monitoring has attracted growing research attention in the context of industry competitiveness, environmental sustainability, and the development of AI-based sensing and analytics technologies [8]. The integration of machine vision, sensor networks, and data-driven analytics into feedlot operations has been associated with improvements in real-time health monitoring, early disease detection, and feeding precision [3]. These approaches have the potential to reduce resource waste and environmental impact through improved feed conversion and waste management, and to support regulatory compliance and traceability in beef supply chains [9,10].

The adoption of these technologies represents a broader process of feedlot modernisation, encompassing not only the integration of new hardware and software but also the transformation of management practices, workforce skills, and decision-making frameworks required to realise their potential in commercial production settings [2,3].

### 1.2. Research Gaps

Despite the rapid advancement of Precision Livestock Farming (PLF) technologies, several critical gaps persist between demonstrated capability and practical, large-scale deployment in commercial feedlot environments. First, in the domain of computer vision and AI, object detection and behavioural recognition models, including YOLO-based architectures [8,11,12], have demonstrated strong performance under controlled conditions. However, their robustness under the full range of real-world feedlot conditions, including dust, variable lighting, extreme weather, and high animal density, remains insufficiently characterised. Most re-identification and individual recognition frameworks have been validated on small, homogeneous datasets that do not reflect the genetic and phenotypic diversity of commercial cattle populations [12]. Second, in the domain of liveweight estimation and growth monitoring, image-based methods such as SCA-ConvNet [13] have shown promising accuracy in controlled settings. However, large-scale commercial validation across diverse breeds, body condition scores, and environmental conditions is still lacking, and the integration of such models into continuous, real-time feedlot management workflows remains an open engineering challenge. Third, in the domain of feed and water intake monitoring, breed-specific differences in feeding behaviour and their influence on the predictive accuracy of remote monitoring technologies, including RFID-linked electronic feeders and accelerometer-based ear tags, have only recently begun to be investigated [14]. Further work is needed to determine how these differences should be accounted for in dry matter intake prediction models before reliable commercial deployment across mixed-breed feedlots can be achieved. Fourth, in the domain of automation and decision support, algorithm-based bunk management systems have demonstrated performance comparable to experienced human operators at commercial scale [15]. However, current systems demonstrate limited adaptability to changes in animal physiology and environmental conditions over the feeding period. Similarly, LLM and RAG-based decision support frameworks for livestock operations [2] remain at an early stage of development, with no published large-scale validation in operational feedlot environments reported to date. Fifth, persistent barriers to adoption, including high capital costs, inadequate rural connectivity, limited digital literacy among farm workers, and sociocultural factors, continue to constrain PLF uptake, particularly in low- and middle-income regions and among small to medium-scale producers [2,16]. Evidence-based interventions addressing both technological readiness and the practical constraints of diverse farming contexts are needed to support equitable and effective adoption.

### 1.3. Objectives of This Review

This review provides a structured synthesis of current technologies, systems, and practices underpinning smart feedlot development within the framework of Precision Livestock Farming (PLF) [16]. It examines the integration of sensors, automation, and data-driven analytical tools in feedlot operations, with a focus on animal health, welfare monitoring, and sustainable management [3]. This review also identifies key implementation challenges, including occlusion in vision-based systems, connectivity constraints in rural environments, and limited adoption in extensive grazing contexts, and proposes research directions to address technological, economic, and ethical gaps. By mapping the current state of the art and identifying emerging trends, this review aims to support researchers and industry stakeholders in evaluating the evidence base, limitations, and deployment requirements of smart feedlot technologies in both confined and extensive livestock systems [16].

## 2. Methodology, Scope, and Scale

This review adopts a structured narrative review methodology, combining a systematic and reproducible literature retrieval process with expert-informed thematic synthesis, conceptual mapping, and text mining to synthesize technological, welfare, and operational advancements in smart feedlot systems. The scope encompasses digital infrastructure, animal welfare monitoring, human–animal interactions, and autonomous technologies, with insights drawn from over 350 peer-reviewed publications and industry reports spanning both academic research and practical system development.

### 2.1. Methodology

This structured narrative review adopts a multi-phase methodology to synthesize scientific research and emerging technological developments related to smart feedlot systems, digital livestock management, animal welfare, and autonomous operations. The approach combines systematic literature retrieval, expert-driven thematic development, conceptual mapping, and quantitative text mining to ensure comprehensive and practically relevant coverage.

#### 2.1.1. Research Context and Author Expertise

The authors have direct experience in the development of computer vision, artificial intelligence, and automated welfare assessment systems for commercial feedlot environments. This applied background informed the scoping and thematic prioritisation of this review, consistent with standard practice in expert-informed narrative reviews [2,3]. However, all substantive claims presented in this review are grounded in peer-reviewed literature, industry reports, and validated technical documentation identified through the systematic search process described below.

#### 2.1.2. Literature Search and Selection Process

A systematic literature search was performed using Web of Science, Scopus, and Google Scholar, supplemented by targeted searches within specialist journals including *Frontiers in Veterinary Science*, *Computers and Electronics in Agriculture*, and *Animals*. Search terms were developed based on established concepts in the literature [17,18,19,20,21]. Key search terms included combinations of: smart feedlot, Precision Livestock Farming (PLF), animal welfare, machine vision, image-based monitoring, autonomous systems, digital agriculture, stockperson interaction, wireless networks, remote sensing, and surveillance systems.

The Boolean search strings in Table 1 were applied across the three primary databases.

Publications were included if they addressed technological, welfare, or operational dimensions of smart feedlot or PLF systems, reported empirical findings or structured reviews, and were published in peer-reviewed journals, conference proceedings, or as technical reports from recognised research institutions. Publications were excluded if they were unavailable in English, lacked methodological transparency, or were primarily opinion pieces without empirical grounding.

The initial search yielded approximately 550 publications, of which over 100 peer-reviewed articles, systematic reviews, and technical reports were retained for in-depth analysis based on their relevance to the technological, welfare, and operational dimensions of smart feedlots, and an additional 150–200 supporting publications, including conference proceedings, project reports, and emerging research outputs, were screened to provide broader context and trend identification.

Of the approximately 350 publications retained for detailed analysis, 117 are formally cited in this review as representative or illustrative references for each thematic area; the remainder informed the thematic synthesis and trend identification without individual citation.

#### 2.1.3. Text Mining and Research Trend Analysis

Text mining and frequency analysis were applied to the collected literature to quantify the prominence of major research areas, following methodologies used in recent bibliometric reviews in PLF and smart farming [22,23]. This analysis confirmed the centrality of: digital infrastructure, wireless connectivity, and sensor deployment [24,25,26]; AI, machine vision, and image-based health and behaviour monitoring [8,19,27,28]; and animal welfare assurance frameworks and human–animal interaction monitoring [19,21,29,30].

**Figure 1:**
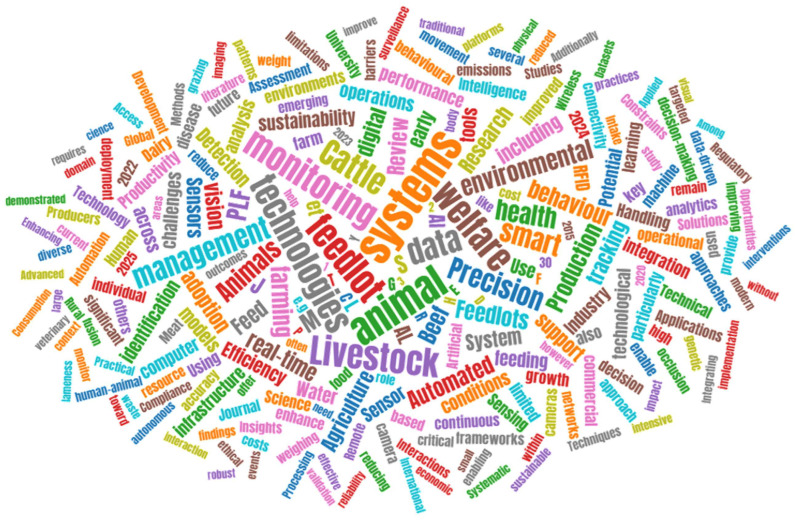
Keyword frequency cloud generated from the titles, abstracts, and author keywords of the approximately 350 publications included in this review. Font size is proportional to keyword frequency across the reviewed corpus.

Figure 1 presents a keyword frequency cloud generated from the titles, abstracts, and author keywords of the approximately 350 publications retained for detailed analysis. Font size is proportional to keyword frequency across the reviewed corpus, providing a visual representation of the dominant research themes identified through the text mining process.

#### 2.1.4. Review Structure and Thematic Coverage

The final structure of this review reflects both the conceptual mind map and prevailing research trends, organized as follows: Digital Agriculture and Smart Feedlot Evolution; Technological Foundations and Infrastructure; Animal Welfare, Human–Animal Interaction, and Monitoring; Enabling Technologies, AI, and Data Analytics; Applications for Health, Welfare, and System Optimization; Implementation Challenges and Industry Trends; and Future Directions toward Autonomous, Welfare-Centric Feedlots.

### 2.2. Scope and Scale

The publications included in this review primarily span the period from 2015 to 2025, reflecting the decade during which Precision Livestock Farming technologies began transitioning from early proof-of-concept demonstrations toward commercially relevant deployments in intensive livestock systems. A smaller number of foundational references extend back to approximately 2000, included where they provide essential historical or conceptual context. Prior to 2015, research in this domain was relatively sparse, largely confined to early RFID deployments, basic sensor studies, and preliminary machine vision investigations. From 2015 onward, the field experienced substantial growth in publication volume, driven by advances in deep learning, the proliferation of affordable IoT hardware, and the maturation of cloud and edge computing infrastructure. The most recent period, 2020 to 2025, has seen the emergence of new research streams including LLM and RAG-based decision support, multi-sensor fusion for individual animal re-identification, and algorithm-based feedlot management automation. This temporal distribution is reflected in the thematic emphasis of this review, with greater depth of coverage allocated to areas where the evidence base has grown most substantially in recent years.

Consistent with a structured narrative review framework, this review synthesises evidence narratively and thematically, drawing from peer-reviewed journal articles, industry reports, and technological case studies published over the past two decades. The scope covers smart feedlot technologies applied to both confined and extensive livestock operations, with a focus on cattle. Key areas of analysis include sensor technologies, RFID systems, automated weighing, AI-based monitoring, and computer vision applications. This review also evaluates barriers to adoption, welfare implications, and system integration challenges, with emphasis on commercially relevant innovations and practical deployment scenarios. In total, approximately 350 academic publications were synthesized thematically, of which 117 are formally cited; the remaining publications informed the broader thematic synthesis and trend identification. Supplementary industry reports and technical documentation were also consulted. The approximated number of key references is shown in Table 2.

## 3. The Digital Agriculture Revolution and Feedlot Systems

**Figure 2:**
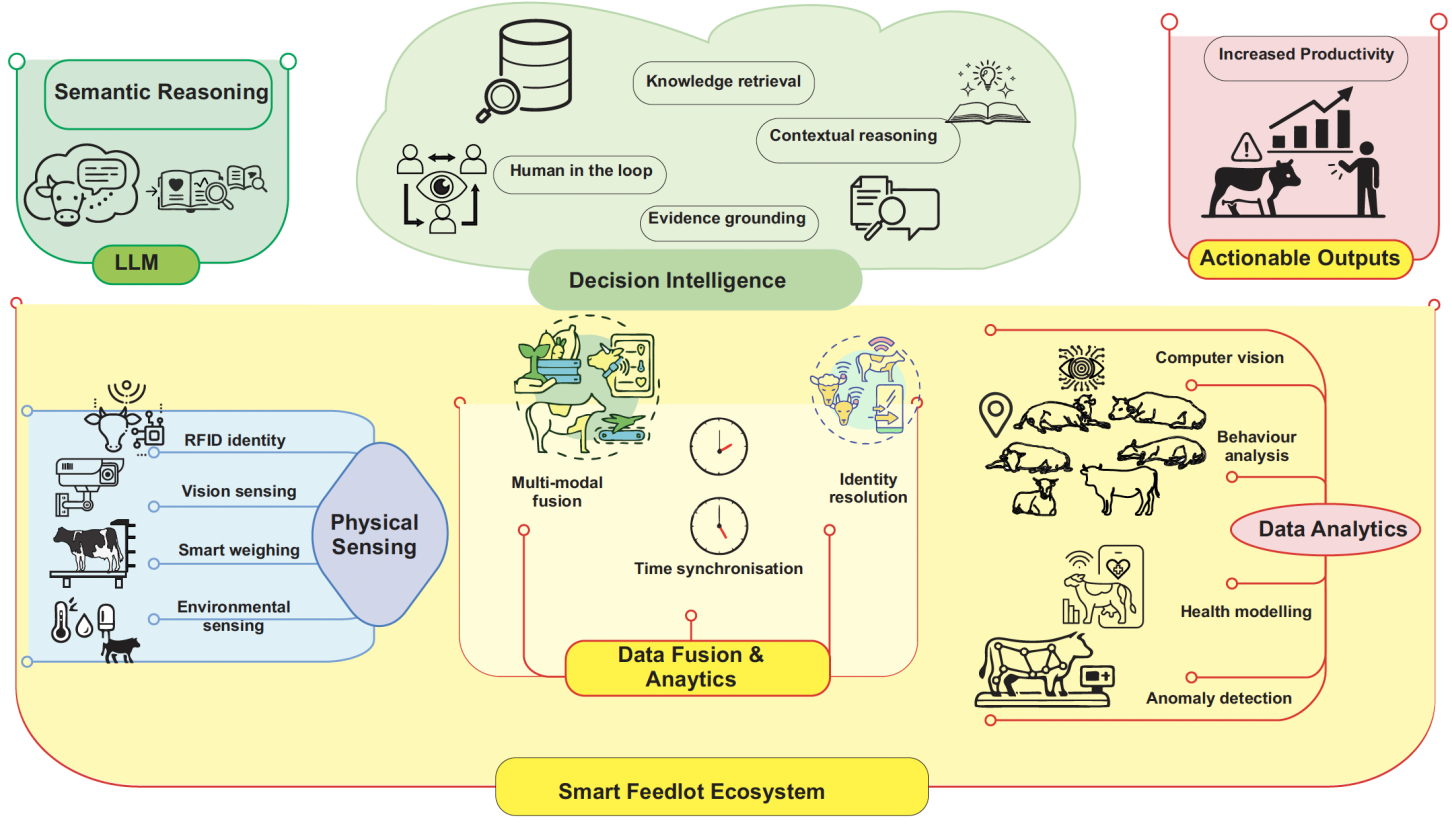
Elements of a Smart Feedlot Ecosystem including: (1) Physical Sensing captures real-time livestock and environmental data through RFID identification, vision sensing, smart weighing, and environmental monitoring; (2) Data Fusion & Analytics integrates multi-modal data streams via time synchronization and identity resolution; (3) Data Analytics applies computer vision, behavior analysis, health modeling, and anomaly detection to extract meaningful insights; and (4) Decision Intelligence leverages large language models (LLMs) for semantic reasoning, combined with knowledge retrieval, contextual reasoning, human-in-the-loop validation, and evidence grounding to generate actionable outputs that enhance feedlot productivity and animal welfare.

**Figure 3:**
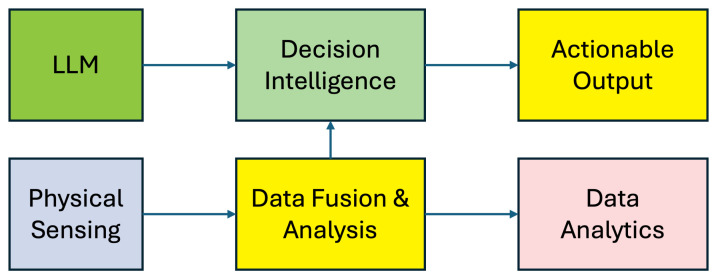
Interaction between elements of a smart feedlot ecosystem.

The emergence of smart feedlots represents a key application of digital agriculture principles within intensive livestock systems, combining automation, real-time data, and precision management to transform traditional feedlot operations. Figure 2 illustrates an overview of different elements in the smart feedlot ecosystem, and Figure 3 illustrates the interaction workflow between these elements.

### 3.1. Precision Livestock Farming (PLF)

PLF encompasses a wide array of technologies and systems designed to optimize livestock management through automation, sensor integration, and data analytics for real-time monitoring, decision support, and targeted interventions. In smart feedlot systems, PLF enables producers to shift from broad, reactive herd management to precise, data-driven individual animal care [3]. A wide range of technologies underpin this transformation. These include automated weighing stations, Radio Frequency Identification (RFID) for animal identification and tracking, body temperature sensors, Geographic Information Systems (GIS) support for evaluating and managing pasture conditions, and unmanned aerial vehicles (UAVs) for remote monitoring of herd behaviour and location (for example [17]). A Geographic Information System (GIS) supports condition monitoring in a feedlot by providing spatial mapping and real-time visualization of animal movement, health patterns, and infrastructure status. By integrating data from sensors, RFID tags, and environmental sources, GIS enables early detection of issues such as overcrowding, abnormal behaviour, or equipment failures. This spatial insight allows for targeted interventions, improved animal welfare, and more efficient feedlot management [46]. More recent innovations, such as virtual fencing, allow producers to manage grazing patterns without physical barriers [37]. Together, these tools not only enhance productivity but also contribute to improved animal welfare, biosecurity, and environmental stewardship.

### 3.2. The Evolution of Precision Livestock Farming (PLF)

The evolution of cattle production has laid the groundwork for the widespread adoption of Precision Livestock Farming (PLF), which represents the latest phase in a long trajectory of system intensification and technological advancement [3,22,47]. PLF encompasses the use of real-time data, automated monitoring, and decision-support tools to optimize animal health, productivity, and welfare. Its rapid emergence builds upon decades of progressive transformation within the beef sector.

Historically, cattle production was extensive, and low-input [23]. Prior to the 20th century, animals grazed freely on open rangelands, with limited human intervention aside from seasonal herding and transport to market [48]. Centralized slaughter operations, such as Chicago’s Union Stock Yards (established in the late 19th century) [49], improved processing efficiency but had little immediate effect on on-farm management.

The mid-20th century marked a pivotal shift toward intensification. Rising global meat demand and surplus grain availability in North America spurred the development of confined feedlot systems [50]. Cattle were finished on grain-based rations, significantly accelerating growth and improving carcass quality. This period also introduced major genetic innovations, often referred to as the breed revolution [51]. British breeds (e.g., Angus, Hereford) became widely used, while continental breeds (e.g., Charolais, Limousin) were imported to enhance growth potential and lean meat yield.

By the 1970s and 1980s, genetic selection was systematized through Expected Progeny Differences (EPDs), while advances in nutrition, feed additives, and veterinary protocols enhanced efficiency and consistency [52]. Feedlots expanded in scale, routinely handling thousands of animals under controlled management conditions [53].

Modern agriculture is undergoing a digital transformation driven by Industry 4.0 technologies: the Internet of Things (IoT), big data, artificial intelligence (AI), cloud computing, and high-speed connectivity such as 4G and 5G [18,24,25]. These technologies collectively define the Agriculture 4.0 paradigm, enabling real-time, automated, and data-driven management across farming systems.

Within this broader digital transition, livestock production has witnessed the rise of Precision Livestock Farming (PLF), a field that adapts Industry 4.0 innovations to the individual monitoring and management of animals. Unlike traditional herd-level approaches, PLF emphasizes continuous, non-invasive, and individual-level monitoring of animals [18]. Through wearable biosensors, RFID-enabled tracking, machine-vision systems, and smart devices, PLF systems can detect early signs of illness, monitor behaviour, and support predictive, data-informed decision-making. These technologies are increasingly powered by AI-based analytics capable of transforming vast datasets into predictive insights.

To support these capabilities, cloud computing and mobile connectivity, especially with the advent of 5G, allow for low-latency data transmission and remote management. For instance, producers can receive alerts when a steer displays signs of fever or reduced mobility, enabling earlier, targeted interventions and improved animal welfare outcomes.

The barriers and opportunities associated with digital transformation in livestock production are well recognised. They point to a critical need to accelerate the adoption of Digital Agriculture and Industry 4.0 (I4.0) solutions [2,16]. The growing complexity of livestock systems, coupled with escalating demands for productivity, animal welfare, and environmental sustainability, renders traditional management approaches increasingly inadequate. Digital technologies and I4.0 frameworks offer the means to address these pressures through real-time monitoring, predictive decision-making, and more efficient resource use. However, realising their full potential will require concerted effort. Implementation costs must be reduced, reliable digital infrastructure expanded, and producers equipped with the necessary digital skills and decision-support tools. Policy frameworks that facilitate standardisation, interoperability, and open data exchange across platforms and devices are equally important. Such coordinated efforts will be instrumental in scaling digital solutions effectively and ensuring they contribute meaningfully to improved animal welfare, enhanced biosecurity, and the broader goal of sustainable livestock production.

### 3.3. Industry 4.0 and Digital Agriculture in Livestock

Building on the evolution of Precision Livestock Farming (PLF) and its role in modern feedlot operations, these technological advancements are not occurring in isolation [16]. They are part of a broader global shift towards more sustainable and responsible food production systems. The increasing emphasis on sustainable agriculture reflects the urgent need to balance livestock productivity with the protection of natural resources and ecosystems [38]. Intensive livestock systems, such as feedlots, are at the forefront of this challenge, facing heightened scrutiny over their environmental footprint, including greenhouse gas emissions, water consumption, nutrient runoff, and land degradation. These pressures are further exacerbated by the impacts of climate change, biodiversity loss, and rising public demand for transparency and environmental accountability across the food supply chain.

Achieving sustainability in feedlot operations demands a system-level approach that integrates ecological, technological, and social dimensions [54]. Smart feedlot systems enabled by PLF directly support this goal: precision feeding reduces feed waste and enteric methane emissions per unit of output, IoT-based water monitoring optimises consumption and detects leaks, and real-time environmental sensors maintain housing conditions that mitigate disease risk and pollution. Socioecological frameworks [55] help evaluate the trade-offs among productivity, welfare, and environmental outcomes, and are particularly relevant in contexts such as Australia, where livestock emissions and water stress require careful balancing of industry growth with environmental responsibility. The sustainability implications of PLF technologies, including their role in reducing methane emissions, optimising resource use, and supporting socioecological assessment frameworks, are discussed in detail in Section 9.5.

### 3.4. From Traditional to Precision Systems

In conventional feedlot systems, especially in extensive grazing environments, economic and labour constraints make it impractical to assess the health and performance of each animal individually. This limitation is becoming increasingly problematic as herd sizes expand and consumer expectations for ethical and transparent food production rise. Precision Livestock Farming (PLF) has emerged as a transformative response to these challenges. By leveraging digital technologies, PLF enables real-time, continuous monitoring of individual animals, thus facilitating early detection of health issues, behavioural anomalies, and performance deviations. This shift represents a fundamental change in livestock management philosophy, from reactive, herd-level intervention to proactive, individual-level care.

This transition is particularly evident in feedlots, where animals are housed in controlled environments that are more conducive to the deployment of digital technologies compared to extensive paddock systems. Table 3 outlines the key operational and environmental differences between feedlots and paddocks, illustrating why feedlots are better positioned to benefit from Precision Livestock Farming (PLF) technologies.

This transition from reactive, herd-level intervention to proactive, individually responsive management represents the operational core of feedlot modernisation, and its realisation depends as much on producer readiness and workforce capability as on the availability of the underlying technologies.

## 4. Definition of Smart Feedlots

A smart feedlot is a livestock production system enhanced through the integration of advanced digital technologies, such as sensor networks, automation, and artificial intelligence, to enable real-time, individualized management of animal health, welfare, productivity, and environmental sustainability. Operating within the broader framework of Precision Livestock Farming (PLF), smart feedlots emphasize continuous, data-driven decision-making to optimize both herd and individual animal outcomes.

### 4.1. The Role of Smart Feedlots in Modern Livestock Production

A smart feedlot substantially enhances animal welfare through precise, early detection and mitigation of health issues. Technologies such as infrared thermography have proven effective in identifying elevated body temperatures, a common early indicator of illness, well before visible clinical symptoms emerge [24]. This capability allows for prompt interventions that significantly reduce animal suffering (needs a reference). Additionally, the use of automated weighing and sorting systems [46] minimizes stressful handling interactions by limiting direct human–animal contact, further promoting animal comfort and welfare (needs a reference). Furthermore, Biosecurity protocols are considerably strengthened in smart feedlots through automated tracking and sorting technologies [56]. Radio Frequency Identification (RFID) systems, when integrated with automated sorting gates [55], rapidly identify and isolate at-risk or symptomatic animals, thereby significantly curbing disease transmission and minimizing outbreaks (needs a reference). Complementing these measures, environmental sensor networks continuously monitor critical parameters such as ammonia, humidity, temperature, and CO_2_ levels. Maintaining optimal environmental conditions effectively prevents pathogen proliferation, enhancing overall biosecurity [57]. Finally, smart feedlots support environmental sustainability by optimizing resource usage, thereby reducing environmental impacts. Automated precision feeding systems minimize feed waste, enhance feed conversion efficiency, and consequently lower greenhouse gas emissions per unit of beef produced. Research indicates that precision feeding technologies can improve feed conversion ratios by up to 10%, directly translating into decreased methane emissions and improved environmental performance [58]. Additionally, smart water troughs and IoT-enabled water management systems [41] facilitate real-time monitoring of water consumption, further reducing resource wastage and nutrient runoff, thus positively impacting sustainability goals.

### 4.2. Global Trends in Livestock Demand and Production

Over the past four decades, global dietary patterns have shifted significantly, with a marked rise in the consumption of animal products [59]. This surge is primarily driven by the economic growth of emerging markets, particularly in South America and Asia, where increasing incomes and urbanization have spurred higher demand for meat and dairy [60,61,62]. In response, livestock production systems have scaled up dramatically, transitioning from smallholder or subsistence farming to intensive, commercialized operations [2,59]. These changes have placed new pressures on producers to manage larger herds more efficiently, while also addressing sustainability, animal welfare, and traceability concerns.

## 5. Foundations for Smart Feedlot Development

### 5.1. Technological Innovations in Feedlot Systems: Foundations for Precision Livestock Farming (PLF)

The intensification of beef production has been driven by continuous technological innovation, progressively laying the foundation for the adoption of Precision Livestock Farming (PLF) tools. PLF, characterized by real-time monitoring, automated control, and data-driven decision-making, represents the logical extension of these advancements, offering new opportunities to enhance productivity, efficiency, animal welfare, and environmental sustainability within modern feedlot systems. In the following sub-sections, a categorical classification of key technological domains underpinning this transformation will be presented. Subsequently, each technology will be examined in greater detail, highlighting its role in advancing precision-based feedlot management.

#### 5.1.1. Nutrition and Feed Technologies

Advances in feeding science have transformed rations from basic grain mixes to highly optimized total mixed rations (TMR) incorporating forages, grains, minerals, and targeted supplements [63]. High-energy processing methods, such as steam-flaking, improve starch availability and feed conversion efficiency. Nutritional additives, including vitamin-mineral premixes, ionophores, and probiotics, further enhance rumen function and animal performance [64]. Computer modelling and real-time data are commonly used by specialized feedlot nutritionists to adjust diets based on market conditions and animal growth metrics [65], exemplifying early-stage PLF applications in nutrition management.

#### 5.1.2. Genetic Improvement

Genetic advancements have played a central role in improving production efficiency [66]. Modern feedlots increasingly rely on specialized beef breeds and crossbreds, selected for traits such as rapid growth, carcass quality, and disease resistance [67]. The widespread adoption of artificial insemination and genomic selection has accelerated gains in average daily weight gain and feed efficiency [68]. The ability to integrate genetic information with real-time performance data exemplifies the potential of PLF to further optimize genetic progress within feedlot systems [69].

#### 5.1.3. Animal Health and Welfare

Preventive health programs, including vaccination, deworming, and regular monitoring, are now standard practice [70]. The emergence of PLF-enabled health tools, such as wearable sensors, automated cameras, and behavioural monitoring algorithms, provides early detection of illness through continuous surveillance of individual animals [31]. Infrastructure improvements, including shaded pens, sprinkler systems for heat stress mitigation, and welfare accreditation schemes (e.g., Australia’s National Feedlot Accreditation Scheme), further reflect the sector’s commitment to health and welfare, now increasingly supported by digital technologies [71].

#### 5.1.4. Automation and Data Analytics

Automation and data analytics constitute a fundamental component of modern feedlot operations and are central to the advancement of Precision Livestock Farming (PLF). As this topic forms the primary focus of the present review, a detailed examination of the underlying technologies, their applications, and current developments will be presented in the subsequent sections.

#### 5.1.5. Environmental Management

As environmental sustainability becomes a critical priority [42], technological solutions targeting emissions reduction and nutrient management [72] have been widely adopted. Manure treatment systems [73], including lagoons, composting, and biogas capture, mitigate environmental impacts [74] while generating valuable by-products [75]. Feedlot design incorporates features such as concrete pads and engineered drainage to prevent nutrient leaching. PLF tools now support precision environmental monitoring, while dietary interventions, including methane-reducing feed additives [43], demonstrate the sector’s capacity to contribute to climate-smart production. Notably, lifecycle analyses indicate that feedlot finishing, through improved feed efficiency and shortened finishing periods, can reduce per-unit greenhouse gas emissions compared to extensive pasture-based systems [44].

Collectively, these innovations underscore the feedlot sector’s technological trajectory, with PLF emerging as a key enabler of more efficient, welfare-conscious, and environmentally responsible beef production.

### 5.2. Integration with Grazing and Confinement Systems

Grazing and confinement systems are two primary methods of cattle management. Grazing systems allow animals to feed on natural pastures, promoting natural behaviour and often reducing feed costs, while confinement systems keep cattle in controlled environments where feed, health, and growth can be closely monitored and managed [76]. Each system has advantages and challenges, and many operations use a combination to balance animal welfare, productivity, and resource efficiency.

When combined with grazing and confinement systems, this infrastructure enables seamless tracking and management of animals across different environments, improving feed efficiency, welfare, and operational flexibility. Integration allows for real-time data-driven decisions that balance pasture utilization with feedlot performance, enhancing overall productivity and sustainability.

## 6. Human–Animal Interactions and AI Monitoring

### 6.1. Stockperson Cattle Interaction

According to Rushen, et al. [29], the knowledge, technical skills, and attitudes of those caring for livestock directly influence housing decisions, feeding practices, disease management, and routine procedures such as dehorning or tail docking. A growing body of research highlights the significant impact of human–animal interactions on cattle welfare, with poor handling practices contributing to stress and reduced productivity. As emphasized by Hemsworth et al. [30], effective stockmanship is fundamental to ensuring animal welfare. Although traditionally underexplored [77], the influence of stock people is now gaining recognition, particularly in systems where animals depend heavily on human care. The frequency and nature of human–animal contact vary across production systems, with dairy cattle and veal calves experiencing more frequent handling than extensively raised beef cattle, which has shaped the focus of research in this area.

### 6.2. Stockperson Behaviour

While extensive research has explored stockperson behaviour and its impact on animals in production systems with frequent handling, such as pigs, dairy cattle, and veal calves, comparable studies in suckling beef cattle are scarce due to less frequent human contact. A recent study [78] addressed this gap by developing a tool to assess stockperson behaviour during the routine but infrequent weighing of beef calves on commercial farms. Destrez, et al. [78] recorded and categorized 230 handling sequences across six farms, using multiple correspondence analysis and hierarchical clustering. Three distinct handling styles emerged: predominantly negative practices (e.g., hitting, tail twisting), neutral practices (e.g., talking, pushing), and predominantly positive practices (e.g., gentle touching, absence of hitting or twisting). Positive handling was associated with shorter weighing times and reduced calf vocalizations, indicating lower stress levels. These findings align with established research in intensively managed species, highlighting that even infrequent handling events can significantly influence the human–animal relationship in beef cattle production. Consequently, monitoring stockperson behaviour during such events offers a practical approach to identifying suboptimal human–animal relationships in extensive beef systems.

Traditional methods of monitoring these interactions often rely on intermittent observations, which may be subject to human bias and limited in scope. Advancements in artificial intelligence (AI) and computer vision technologies offer promising solutions for continuous and objective monitoring of human–animal interactions in feedlot environments. Wurtz, et al. [27] conducted a systematic review highlighting the potential of machine vision systems to automatically record and analyse the behaviour of indoor-housed farm animals, facilitating large-scale phenotyping and welfare assessment.

Implementing AI-based monitoring systems in feedlots can provide several benefits:

- Continuous Surveillance: AI systems can operate 24/7, capturing real-time data on stockperson–animal interactions, thereby reducing the likelihood of unnoticed negative behaviours.
- Objective Assessment: By minimizing human subjectivity, these technologies ensure consistent evaluation of handling practices, contributing to more accurate welfare assessments.
- Data-Driven Feedback: The collected data can inform targeted training programs for stockpersons, promoting best practices and enhancing overall animal welfare.

Furthermore, integrating wearable sensors into livestock management has shown promise in monitoring animal health and behaviour. Neethirajan [79] discusses recent advances in wearable sensor technologies that enable real-time health monitoring, which can be instrumental in detecting stress responses resulting from human interactions.

Physiological indicators, such as heart rate variability and infrared thermography, have also been utilized to assess stress levels in animals. Schillings et al. [19] explored the application of these non-invasive methods to evaluate autonomic activity in domestic animals, providing valuable insights into their welfare status. Additionally, behavioural indicators like play behaviour have been recognized as measures of positive welfare states. Mintline et al. [80] investigated the effects of disbudding on dairy calves’ play behaviour, suggesting that reductions in such behaviours may reflect compromised welfare.

Incorporating AI technologies for monitoring human–animal interactions aligns with the ethical considerations of modern animal agriculture. Croney et al. [81] discuss the implications of the ethical food movement, emphasizing the role of science and technology in addressing public concerns about animal welfare. By adopting AI-driven monitoring systems, smart feedlot operations can enhance animal welfare, improve productivity, and meet the evolving ethical standards expected by society.

### 6.3. AI-Based Compliance Monitoring: Lessons from Slaughter Facilities for Smart Feedlot Design

The on-farm human–animal interactions discussed in Section 6.1 and Section 6.2 do not occur in isolation, their welfare consequences extend through transport and into slaughter, and the AI monitoring approaches being developed for post-farm settings offer directly transferable lessons for smart feedlot system design. Slaughter facilities represent one of the most advanced contexts for AI-driven compliance monitoring in livestock handling, and the methods validated there provide a concrete reference point for feedlot applications.

Edwards-Callaway et al. [36] evaluated the agreement between AI and human evaluators in assessing cattle handling outcomes across 112 video clips from a commercial UK slaughter plant. The AI system identified key welfare-relevant events including stunning efficacy, electric prod usage, falls, pen crowding, and instances of questionable handling. Agreement between AI and human evaluators was quantified using the Jaccard Similarity Index (JI), also referred to as the Jaccard Similarity Coefficient or Intersection over Union (IoU). The JI is calculated as the ratio of the number of events jointly identified by both evaluators (the intersection) to the total number of events identified by either evaluator (the union), expressed formally as follows:

(1) $$JI=\frac{\left|A\cap B\right|}{\left|A\cup B\right|}$$

where *A* and *B* represent the sets of events identified by the AI system and the human evaluator respectively. The index ranges from 0 to 1, where a value of 0 indicates no overlap between the two sets of identifications and a value of 1 indicates perfect agreement. In practice, values above 0.75 are generally considered to reflect strong agreement, values between 0.50 and 0.75 indicate moderate agreement, and values below 0.50 suggest limited correspondence between evaluators.

In the study by Edwards-Callaway et al. [36], high agreement was achieved for stunning efficacy, electric prod usage, and falls (JI > 0.90), with consistent performance for pen crowding (JI > 0.80). Agreement was moderate for questionable handling events (JI ≥ 0.50), reflecting the greater subjectivity involved in defining this category, though the AI system remained effective at flagging such events for human review.

It should be noted that the Jaccard Similarity Index has several limitations in this context. First, the index treats all events equally regardless of their welfare significance, meaning that a missed stunning event and a missed instance of pen crowding contribute equally to the score despite their very different welfare implications. Second, the JI is sensitive to the total number of events in the dataset; in low-frequency event categories such as falls, a small number of disagreements can disproportionately reduce the index value. Third, the index does not distinguish between false positives and false negatives, which may be important when evaluating AI systems intended for regulatory compliance monitoring, where the consequences of missing a welfare violation differ substantially from those of incorrectly flagging a non-violation. These limitations should be considered when interpreting JI values as a measure of AI-human evaluator agreement in animal welfare assessment contexts.

These findings are directly relevant to smart feedlot monitoring. The same computer vision and compliance-tracking architecture demonstrated at slaughter facilities can be adapted to feedlot pens and races to provide continuous, objective surveillance of stockperson–animal interactions, monitoring adherence to handling protocols, appropriate use of equipment, and compliance with animal welfare standards [79]. Integrating such AI-driven compliance monitoring into the broader smart feedlot framework, alongside the health, behaviour, and performance monitoring systems described elsewhere in this review, would extend real-time welfare assurance across the full handling continuum, from daily pen management through to yard work and pre-transport procedures.

## 7. Smart Feedlot Infrastructure and Digital Technologies

A Smart Feedlot, built on the principles of Precision Livestock Farming (PLF) [17], represents an integrated technological framework aimed at improving animal welfare, productivity, and resource efficiency. These systems combine sensing technologies, automation, and data analytics to enable large-scale, individualized monitoring and data-driven decision-making. At the core of this approach are RFID tagging systems, which uniquely identify each animal, allowing for continuous tracking of health status, weight progression, and behavioural patterns. Automated weighing stations or smart scales [55], typically positioned at water or feed points, record animal weights without human intervention, providing reliable data to calculate Average Daily Gain. ADG is a critical indicator of growth performance and overall health.

To enable precise monitoring of feed and water intake, automated troughs (for both water and feed) [82] record consumption levels and alert managers to deviations that may indicate illness. These systems are complemented by environmental sensors that continuously monitor key parameters such as temperature, humidity, ammonia, CO_2_ levels, and wind speed, helping to maintain optimal comfort conditions for the animals. Wearable activity trackers and camera-based monitoring systems, including CCTV and computer vision, further enhance welfare management by detecting behavioural abnormalities, postural changes, gait irregularities, and early signs of lameness. In some cases, infrared thermography is also integrated to noninvasively identify elevated body temperatures, swelling, or other health indicators.

IoT gateways and edge devices collect and preprocess distributed sensor data, which is then transmitted to a centralized data management platform [24]. This platform aggregates live feedlot data and historical trends, providing operators with real-time visualizations and actionable insights. To further enhance decision-making, cloud-based AI and machine learning algorithms are applied for predictive modelling, anomaly detection, and behavioural pattern recognition. In parallel, automated animal sorting and drafting systems can leverage real-time data to route animals for veterinary care, market preparation, or tailored nutritional programs.

An emerging frontier in smart feedlots is the application of Retrieval-Augmented Generation (RAG) and Large Language Models (LLMs) [79]. These AI technologies enable advanced query handling over feedlot data, allowing users to ask natural language questions such as, “Which animals showed a drop in feed intake and weight gain over the past three days?” RAG systems integrate LLMs with structured databases and historical records to generate accurate, insightful responses, significantly enhancing user interaction and decision support. When integrated with livestock production software, these tools facilitate seamless access to health records, medication schedules, and compliance tracking. Collectively, these technologies create a highly responsive, data-driven infrastructure that not only improves operational efficiency but also promotes animal welfare and sustainability in modern livestock management.

### 7.1. Camera-Based and Robotic Systems for Automated Monitoring

Camera-based automation plays an increasingly important role in smart feedlot systems [33], providing real-time monitoring of animal welfare, behaviour, and environmental conditions. Broadly, these technologies can be classified into fixed cameras, mobile camera platforms, and specialised imaging systems:

- **Fixed or stationary cameras**, such as CCTV systems, are strategically installed throughout feedlots to provide continuous visual monitoring of animal behaviour, posture, and movement patterns.
- **Mobile camera platforms**, including drones and robotic systems, offer flexible surveillance capabilities, enabling targeted inspection of animals, infrastructure, or specific zones.
- **Specialised cameras**, such as infrared or multispectral imaging systems, enhance detection of subtle health and environmental indicators, including body temperature, inflammation, or feed distribution anomalies.

Robotic systems, though still emerging in feedlot applications, represent a promising extension of mobile monitoring technologies. Ground-based robotic units equipped with cameras and sensors can autonomously navigate pens or alleyways, performing close-range inspections, health assessments, or infrastructure checks with minimal human intervention. These systems are the subject of ongoing research in precision livestock farming, with the potential to complement both stationary and aerial surveillance approaches.

**Drone-Based Monitoring and Automation**

Among mobile platforms, drone-based approaches have gained considerable attention for their ability to provide rapid, large-area coverage of feedlots. Early drone-based livestock monitoring primarily relied on manual video footage analysis to aid in tasks such as animal counting or disease monitoring in quarantined cattle (see, for example, [83,84]).

Subsequent advances introduced automated image processing techniques, including thresholding, morphological operations, and binary masking for object separation. More sophisticated approaches, such as R-CNN-based object detection, improved accuracy by integrating sliding window techniques and leveraging drone altitude for livestock size estimation.

Equipped with thermal and multispectral imaging systems [85], modern drones can detect early signs of disease, monitor animal movement, and assess the uniformity of feed distribution. By reducing manual labour requirements and improving the accuracy of surveillance data, drone technology enhances decision-making, optimises resource allocation, and contributes to improved animal welfare and sustainability in feedlot operations.

A notable example is the work of Alanezi et al. [83], who explored UAV applications in livestock management, focusing on animal detection, counting, and tracking. Their research highlights both the challenges and opportunities associated with drone-based monitoring, underlining its potential to transform livestock management through improved efficiency and sustainability.

While Section 7.1 highlighted the role of camera-based and aerial imaging technologies in livestock observation, these tools alone are insufficient without intelligent systems capable of interpreting the vast volumes of visual and sensor data they generate. The following section explores how machine learning and computer vision algorithms transform raw imagery and sensor streams into actionable insights, providing the foundation for data-driven decision-making in smart feedlots.

### 7.2. Vision-Based Monitoring and Machine Learning for Behavioural Analysis

Machine learning and computer vision systems have become foundational to the advancement of smart feedlots, particularly in animal identification and behaviour monitoring [11]. A key application is the integration of camera-based visual tracking with RFID matching, where animals are equipped with RFID tags for unique identification, and their movements are continuously monitored by strategically placed cameras [12,13]. This hybrid setup overcomes limitations of RFID alone—such as tag collisions or signal dropouts—by reinforcing identification accuracy and enabling non-intrusive, real-time tracking of individual animals.

Recent research highlights the growing sophistication of vision-based analytics [8]. Norton et al. [20] investigated image and sound processing to build digital animal representations, while Milan et al. [86] used machine learning algorithms to monitor physiological and behavioural markers in dairy cattle. Astill et al. [28] demonstrated the integration of smart sensors with big data and IoT platforms in poultry operations. Dominiak and Kristensen [34] contributed frameworks for prioritizing herd alerts using probabilistic models, which have implications for behaviour-driven diagnostics.

In cattle identification, traditional ML models (e.g., SVM, KNN, ANN) have been used with handcrafted features, while recent deep learning models (e.g., CNN, YOLO, Faster R-CNN) offer end-to-end learning for both detection and identification. A systematic review of 55 studies highlights the transition from ML-based to DL-based approaches, with muzzle prints and coat patterns being dominant features. Challenges include dataset scarcity, environmental variability, and real-time processing constraints [87].

Advancements in deep learning, particularly Convolutional Neural Networks (CNNs) and object detection models such as YOLO, enable rapid processing of video feeds to recognize and classify animals based on physical traits. Such models, trained on diverse datasets with annotated images under varying environmental conditions, allow for robust animal identification even in complex or crowded feedlot settings.

Beyond identification, vision-based AI systems support automated behavioural profiling, including gait tracking, rest/activity cycles, and feeding behaviour. This facilitates anomaly detection, whereby deviations from normal patterns—such as signs of lameness, reduced activity, or weight loss—can be flagged early. These approaches reduce reliance on labour-intensive monitoring and contribute to proactive health management and welfare enhancement.

Despite these advances, several important limitations warrant critical attention. First, most studies validating YOLO-based and CNN-based detection models have been conducted under controlled or semi-controlled conditions. Their performance metrics may not generalise to commercial feedlot environments, where variable lighting, dust, extreme weather, and high animal density are common. Second, there is a notable absence of standardised benchmarking across studies: differences in dataset composition, annotation protocols, and evaluation metrics make direct comparison of reported accuracies unreliable. Third, most individual re-identification frameworks have been validated on small, phenotypically homogeneous datasets that do not reflect the genetic and physical diversity of mixed-breed commercial cattle populations, limiting the transferability of reported performance to real-world deployment. Fourth, while behavioural anomaly detection is widely reported as a capability of these systems, few studies provide prospective clinical validation, that is, confirmation that algorithmically flagged anomalies correspond to verified health events with acceptable sensitivity and specificity under commercial conditions.

### 7.3. Artificial Intelligence Frameworks and Decision Support Systems

While machine learning powers specific functions like image classification, artificial intelligence (AI) more broadly encompasses a suite of computational frameworks that support predictive analytics, decision-making, and operational optimization in feedlot systems. These systems are increasingly tasked with integrating behavioural, environmental, and physiological data to generate real-time insights and recommendations.

AI is at the core of Decision Support Systems (DSS), which assist farm operators in making data-driven choices on feeding schedules, breeding strategies, and health interventions [88,89]. These systems utilize structured and unstructured data from diverse sensors and historical records to guide operational strategies under uncertainty.

Emerging AI-driven tools such as digital twins and simulation-based controls represent the next frontier. These tools create dynamic virtual models of individual animals or entire feedlot operations, enabling what-if analyses, scenario testing, and real-time feedback loops to optimize animal management. The integration of such tools not only increases productivity but also supports compliance with ethical and regulatory frameworks.

Overall, AI frameworks extend beyond behaviour monitoring to enable system-level intelligence, bridging the gap between raw sensor data and strategic farm decision-making.

Despite the growing interest in AI-driven decision support for feedlot management, the majority of published DSS frameworks have been validated using retrospective or simulated datasets rather than through prospective deployment under commercial conditions, and standardised evaluation criteria remain absent, rendering cross-study comparison largely impractical. Digital twin and simulation-based platforms, while conceptually promising, remain almost exclusively at the proof-of-concept stage, with validations typically limited to single-site, single-breed scenarios and short observation periods that do not capture the longitudinal variability in commercial feedlot operations. More broadly, a pervasive weakness across the AI-DSS literature is the limited transparency regarding model assumptions, training data provenance, and sensitivity to upstream sensor noise and missing data, factors that collectively constrain confidence in the generalisability and real-world reliability of reported outcomes.

### 7.4. Hybrid Systems: Sensor Fusion for Enhanced Tracking and Diagnostics

Building upon the capabilities of both computer vision and AI, current research increasingly focuses on hybrid systems that integrate multiple sensing modalities—especially combining visual data (camera-based) with non-visual data such as RFID, acoustic signals, or thermal imaging. These sensor fusion strategies aim to improve both the granularity and reliability of animal tracking and health diagnostics [90].

One promising development involves the adaptation of OSNet-EIN, originally designed for human re-identification, to livestock tracking through transfer learning. The model demonstrated strong cross-domain adaptability, showing high accuracy in matching animals across different camera views. A comparative evaluation of Re-ID architectures using Rank-1 accuracy further helped identify optimal approaches for livestock-specific scenarios.

Gait analysis is emerging as a non-invasive tool for diagnosing musculoskeletal disorders in cattle. Since health conditions often cause compensatory changes in posture and locomotion, automated step counting and motion pattern recognition can serve as early indicators of disease [91]. However, real-world implementation requires improvements in model robustness to withstand variable lighting, occlusions, and environmental disruptions commonly found in feedlots.

Despite the progress in individual components, integrating these into scalable, real-time hybrid systems remains a major challenge. Synchronizing data streams, ensuring temporal alignment, and managing computational overhead are active areas of research. Nonetheless, the long-term vision is a fully automated, sensor-integrated platform capable of continuous animal monitoring, individualized health diagnostics, and adaptive intervention strategies.

### 7.5. Rumen Devices

Rumen devices, such as boluses, capsule sensors, and RFID trackers, are ingestible technologies used in livestock to monitor temperature, pH, digestion, and activity for improved health and nutrition management [65]. These IoT-enabled sensors [24] provide real-time data for early disease detection, feed optimization, and reproductive monitoring, enhancing precision livestock farming. While they offer benefits like reduced antibiotic use and increased efficiency, challenges include device retention, battery life, and cost. These technologies play a crucial role in data-driven livestock management, improving productivity and animal welfare.

Some of the technologies used are similar for broiler/laying hens and livestock, including RFID, cameras, and microphones for automated monitoring. Li et al. [92] indicated that RFID and cameras are the most widely used, with RFID dominating overall applications, particularly in behaviour tracking and identification. Cameras are also highly utilized, especially for monitoring activities, but have fewer commercially tested studies. Microphones, while used, are the least popular, mainly applied for weight and activity tracking. This suggests that RFID and cameras are the preferred technologies for automated welfare and behaviour monitoring across different animal systems. Technologies used during transport or at slaughter are beyond the scope of the current work and can be found elsewhere (e.g., [32]).

### 7.6. Animal Movement Tracking

Some of the previously mentioned technologies, e.g., drones, are useful to track animal movement. Movement-related and spatial monitoring technologies in livestock utilize advanced tracking systems to assess animal behaviour, welfare, and health. Key technologies include three-axis accelerometers, which monitor physical movement such as step count, lying bouts, and activity levels, providing early detection of lameness, illness, and oestrus. Radio-frequency identification (RFID) systems track feeding [14] and watering behaviour, while real-time location systems (RTLS) and GPS-based tracking monitor spatial behaviour, movement patterns, and social interactions within pens or pastures. These technologies enable early disease detection, welfare assessment, and optimization of livestock management, though challenges such as data processing, cost, and sensor durability remain critical for widespread adoption [56].

Some of these sensors, such as GPS, accelerometers, and pedometers, have limited feasibility due to cost, short battery life, and lack of supporting data.

Despite these advances, the evidence base for movement tracking technologies presents notable limitations. Most validation studies have been conducted on small, homogeneous cohorts under controlled research conditions, and the accuracy of accelerometer- and GPS-based systems in detecting behavioural events such as lameness onset varies considerably across studies, with reported sensitivity and specificity values ranging widely depending on sensor placement, algorithm choice, and breed characteristics. Furthermore, the absence of standardised protocols for sensor calibration, data preprocessing, and outcome reporting across the movement tracking literature makes it difficult to draw reliable conclusions about the comparative effectiveness of competing technologies or to establish evidence-based guidelines for their adoption.

### 7.7. Feed/Water Intake Monitoring

Feed and water intake monitoring in livestock relies on the combined use of several sensors and technologies [8,15]. For example, RFID-based systems and automated sensors can be used to track feeding and drinking behaviour, providing valuable insights into animal health and performance [14,93,94]. Systems like GrowSafe use RFID ear tags and sensors near feed bunks to record feeding duration and frequency, while Insentec and AniTrace employ collar-mounted transponders and UHF RFID for real-time intake monitoring. These technologies help detect early signs of disease, such as bovine respiratory disease (BRD), by identifying reductions in feed and water intake before visible symptoms appear. Despite their benefits, challenges include animal acclimation, cost, and data interpretation for large-scale commercial use. GrowSafe Beef™ faces adoption challenges due to high costs and discrepancies with veterinary findings [95].

The evidence base for feed and water intake monitoring technologies presents several unresolved tensions. Studies using RFID-based systems such as GrowSafe and Insentec report strong correlations between recorded intake events and manually verified consumption. However, these systems have been predominantly validated in research or feedlot trial settings with controlled access infrastructure, and their performance under commercial-scale, multi-breed, and mixed-sex conditions is less well characterised. A further methodological concern is the inconsistency in outcome measures across studies: some report feeding duration and frequency, others estimate dry matter intake directly, and comparability across these metrics is rarely addressed. Luke et al. [14] demonstrated breed-specific differences in feeding behaviour that materially affect the predictive accuracy of remote monitoring technologies, yet breed is infrequently treated as a covariate in intake monitoring studies. The economic and operational costs of animal acclimation, a prerequisite for reliable data capture in many of these systems, are also largely absent from published cost-benefit analyses, making return-on-investment estimates difficult to interpret.

### 7.8. Sensor Deployment and Wireless Network Architectures

Feedlot sensor deployment and wireless networks face key challenges, including harsh environmental conditions like dust, moisture, and animal interference that can damage equipment. The large, open area complicates achieving reliable wireless coverage, while signal obstruction from structures and animals further weakens connectivity. Power supply limitations demand efficient energy solutions for remote sensors. High device density requires scalable networks to handle data volume without congestion. Ensuring data reliability and low latency is critical but difficult over unstable links. Cost constraints limit advanced infrastructure investments, and integrating diverse devices demands ongoing maintenance. Additionally, securing wireless communications in an open environment poses significant challenges.

**Multi-hop connectivity**: La et al. [96] have explored energy-efficient multi-hop broadcasting in dense Wireless Sensor Networks (WSNs), highlighting key limitations in traditional approaches. MPR-based (Multipoint Relay) mechanisms, commonly used for relay selection, have demonstrated suboptimal performance in high-density settings. In contrast, the Multicast Protocol for Low-power and Lossy Networks (MPL), which leverages the Trickle algorithm, has shown improved efficiency. However, its reliance on overhearing retransmissions poses challenges for low-duty-cycle MAC protocols, where nodes periodically sleep to conserve energy.

To overcome these constraints, La et al. [96] proposed the Beacon-based Forwarding Tree (BFT), a broadcast scheme tailored to beacon-enabled IEEE 802.15.4 networks [97]. BFT maintains network coverage and achieves comparable performance to MPL while better aligning with duty-cycled communication. Experimental evaluations on a Contiki testbed reveal that BFT offers predictable energy consumption, low power use for leaf nodes, and minimal packet loss, particularly under varying broadcast intensities. These findings suggest that BFT is a promising alternative for energy-constrained WSN deployments. Table 4 outlines the difference between Single-Hop and Multi-Hop connectivity.

A study of smart trough systems by Jacobs, et al. [98] evaluated how well different groups of beef cattle adapt to the Super SmartFeed (SSF) precision supplementation system. Adaptation was measured by whether animals consumed more supplements than the group average during training. The results showed that adaptation rates varied by cattle type: 45% of suckling calves, 62% of weaned steers, and 73% of replacement heifers successfully adapted. These findings suggest that the production stage influences how effectively cattle can adopt precision feeding technologies.

### 7.9. Data Analytics and Management Platforms

Flores et al. [88] developed a decision-support modelling tool to project future cattle growth in feedlots using a low-dimensional dataset available at the start of feeding. The tool applies statistical learning methods to project individual animal growth trajectories, group animals by trait similarity for more efficient pen assignments, and estimate optimal sale timing based on projected costs and market prices. The system was designed for small to medium-scale operations and does not require specialised technical expertise. The system was tested on a farm in Sonora, Mexico, where it successfully predicted growth patterns, matched past grouping decisions, and helped identify ways to increase profits.

**Big Data and Cloud Pipelines vs. Edge Processing**: Big data pipelines and cloud processing offer powerful centralized data storage and advanced analytics with scalable resources, ideal for handling massive datasets and complex decision-making. In contrast, edge processing [25] performs data analysis locally near the data source, enabling faster, real-time decisions with reduced latency and lower bandwidth use. While cloud solutions excel in depth and scale of analysis, edge processing is better suited for time-sensitive applications and environments with connectivity constraints. Choosing between them depends on factors like data volume, latency requirements, infrastructure costs, and the need for immediate insights.

## 8. Applications for Animal Health, Welfare, and Monitoring in Feedlots

### 8.1. Applications of Computer Vision and Occlusion Management

Within the domain of Precision Livestock Farming (PLF), three-dimensional (3D) computer vision has demonstrated significant potential for enhancing growth monitoring in cattle management. Wang et al. [99], conducted in accordance with the PRISMA 2020 guidelines, identified and analysed 47 eligible studies sourced from the Web of Science database. Each study was evaluated individually, with outcomes extracted using a structured data collection form and synthesized through pivot analysis.

Their findings indicated that 3D body measurement tasks frequently underpin broader applications in cattle growth monitoring. Among the technologies employed, Microsoft Kinect sensors positioned at overhead (nadir) angles were the most used tools for capturing dorsal body features. For 3D data preprocessing, empty scene subtraction emerged as the most effective method for background removal in point cloud data, while clustering and conditional filtering were the predominant techniques for noise reduction.

Wang, et al. [99] also reviewed the application of 3D computer vision in livestock, offering a synthesized overview of data acquisition protocols, a generalised preprocessing pipeline, and a framework for constructing automated multi-task systems for continuous cattle growth monitoring. Additionally, it identifies key variables influencing the performance of such systems and outlines future opportunities for extending these techniques to other livestock species or wild animals. The findings serve as a foundational reference for advancing automated vision-based systems in precision cattle management.

#### 8.1.1. Multi-Camera Systems for Cattle Surveillance

Multi-camera systems reduce the limitations of single-view monitoring and provide comprehensive coverage of animal behaviour and movement. By strategically placing multiple cameras around feedlots or barns, these systems enable continuous tracking, 3D reconstruction, and multi-angle behaviour analysis. This setup is mainly used to enhance the accuracy in identifying individual animals, detecting anomalies such as lameness or aggression, and monitoring feeding patterns. Integration with computer vision algorithms and data fusion techniques can eventually improve reliability, making multi-camera systems a powerful tool for automated, real-time welfare assessment in precision livestock farming.

While these results are promising, several methodological limitations should be noted. The validation sample for hot standard carcass weight prediction comprised only 32 Angus steers, a homogeneous, single-breed cohort that limits the generalisability of the reported R^2^ and RMSE values to diverse commercial populations. The reliance on cross-validation rather than independent holdout testing further constrains confidence in the reported accuracy figures. More broadly, 3D imaging systems of this type require controlled race conditions and consistent animal positioning, and their robustness to the variation in posture, breed conformation, and body condition score encountered across a full commercial intake is not yet established at scale. Wang et al. [73] similarly note that most 3D computer vision studies in this domain have been conducted under constrained experimental conditions, and that large-scale commercial validation across diverse breeds and environmental contexts remains an open challenge.

Camera placement and lens distortion: In multi-camera tracking systems for cattle surveillance, camera placement is a critical factor influencing tracking performance, especially in optical motion capture, which estimates 3D positions of target points through triangulation. Successful triangulation requires that points be visible to at least two cameras and that the camera network configuration ensures both visibility and geometric diversity [100]. Poor placement can lead to low-resolution imagery, increased occlusion, and inaccurate 3D reconstruction. To address these challenges, researchers have proposed quantitative quality metrics that evaluate camera configurations based on both resolution degradation and probabilistic models of dynamic target occlusion. These metrics help automate the design of tracking systems by optimizing camera poses using algorithms such as simulated annealing [101]. Two notable approaches include one based on computing visibility under occlusion and another on maximizing view diversity across target points. Empirical studies and simulations demonstrate the effectiveness of these methods in producing robust, high-accuracy multi-camera setups. Publicly available implementations of these tools further enable their application in automated livestock surveillance, where continuous and precise monitoring is essential for animal welfare assessment.

View warping and cow appearance challenges: Alempijevic, et al. [102] introduced a real-time 3D imaging system deployed in a commercial feedlot to non-invasively estimate key production traits of live cattle, including hip height (cm), subcutaneous fat thickness at the P8 site (mm), and hot standard carcass weight (HSCW, kg). They utilized multiple cameras installed in a conventional cattle race, which was designed to accommodate variations in animal length and breed. They conducted in two parts: Study 1 compared 3D-derived hip height and P8 fat thickness estimates to manual measurements and ultrasound scans in 247 and 219 steers, respectively; Study 2 compared 3D-based predictions of HSCW to abattoir measurements in 32 Angus steers. While hip height was directly extracted from 3D models, P8 fat and HSCW were predicted using Gaussian Process Regression, trained on features derived from the 3D scans. Model performance was evaluated using cross-validation, yielding promising results: for hip height, RMSE = 3.07 cm and R^2^ = 0.69; for P8 fat, RMSE = 2.38 mm, R^2^ = 0.78; and for HSCW, RMSE = 8.15 kg, R^2^ = 0.79. These findings demonstrate the accuracy and robustness of the 3D imaging system for live-animal phenotyping. The system forms the basis of the CattleAssess3D technology, integrating imaging with BeefSpecs to deliver actionable insights for producers. Its feasibility and adaptability support broader adoption in the beef industry as part of precision livestock farming strategies.

#### 8.1.2. Managing Occlusion in Cattle Monitoring: Field Applications

Occlusion presents a significant challenge in vision-based cattle monitoring, arising from both physical and environmental factors, as well as animal behaviour. Physically, occlusion can result from barn structures, feeding equipment, water troughs, or gates that block camera views. Environmental conditions such as poor lighting, dust, or rain can further obscure visibility. In dynamic feedlot environments, movement interference, including rapid shifts in animal position or head turning, complicates consistent detection and tracking [11,12]. Additionally, herd behaviour, such as clustering, mounting, or overlapping in narrow spaces, leads to frequent self-occlusion among animals. These factors reduce the accuracy of image-based systems in identifying individuals and interpreting behaviour, highlighting the need for robust occlusion-aware models and multi-camera configurations in cattle surveillance systems.

#### 8.1.3. Applied Solutions for Occlusion Handling

To the best of the authors’ knowledge, while occlusion handling has not been explicitly addressed for feedlot monitoring, existing methods from other fields can be leveraged to improve visibility, identification, and behavioural assessment despite visual obstructions. One effective approach involves deploying multi-angle camera arrays [103], which provide overlapping fields of view from different perspectives. By covering the monitoring area from multiple directions, these arrays reduce blind spots and increase the likelihood that key body parts remain visible, even when animals cluster or move unpredictably. This setup is particularly effective in raceways, feed bunks, and densely populated pens.

Another promising solution appears to be the integration of depth and thermal imaging [104]. Depth sensors, such as LiDAR or structured light cameras, capture the 3D shape of animals, allowing systems to distinguish between overlapping bodies using spatial cues. Thermal imaging [104] adds another layer of visibility, detecting heat signatures even in low-light or dusty conditions. These modalities help maintain tracking continuity when visual occlusion occurs in RGB camera streams. Based on our experience developing computer vision technologies for feedlots, we have found these environments to be particularly harsh, posing significant challenges to the durability and reliability of most technological systems.

Advances in AI-based re-identification and object tracking [105] also offer powerful tools for occlusion handling. Deep learning models can recognize individual animals based on unique features such as coat patterns, body shape, or movement signatures. These models can re-identify animals after brief occlusions and predict trajectories using temporal context, making tracking more robust in crowded or obstructed scenes.

Finally, we think that data fusion with non-visual sensors, such as RFID and accelerometers [106], greatly enhances system reliability. RFID tags provide definitive animal identity at fixed checkpoints, which can be synchronized with visual data to correct errors. Accelerometers, often worn on collars or ear tags, capture motion patterns that can help differentiate animals and provide continuous monitoring when visual input is degraded. By combining these diverse data sources, PLF systems can overcome the limitations of visual occlusion and deliver more accurate and resilient cattle monitoring solutions.

#### 8.1.4. Remote Diagnostic Technologies for Feedlot Cattle

Rabiee et al. [107] evaluated current and emerging remote diagnostic technologies aimed at identifying ill-health and shy feeding in feedlot cattle. Using a combination of published literature, manufacturers’ data, expert interviews, and a stakeholder survey, this review appraises the practical benefits and limitations of technologies such as rumen sensors for temperature and pH, movement tracking devices, feed and water intake monitoring systems, and rumination activity sensors. While several technologies show promise for early disease detection and welfare improvement, gaps remain in terms of validation under commercial feedlot conditions, cost-benefit analysis, and integration with systems like the National Livestock Identification System (NLIS) in Australia.

They suggest that among the technologies, rumen boluses measuring temperature and pH offer potential for diagnosing diseases like bovine respiratory disease complex and ruminal acidosis, although sensitivity and specificity data are limited. Systems monitoring feed and water intake were recognized by industry stakeholders as the most reliable indicators of animal health, yet their high costs and operational demands hinder wider adoption. Similarly, technologies for tracking cattle movement and rumination require further refinement and validation to ensure practical applicability. Industry feedback emphasized the need for low-cost, durable, and user-friendly solutions that do not disrupt animal welfare.

Overall, while the reviewed technologies present valuable features for advancing health monitoring in feedlots, widespread implementation depends on further performance validation, cost-effectiveness studies, and industry education regarding their benefits. The integration of multiple complementary technologies, despite potential cost barriers, may offer a more comprehensive approach to improving feedlot cattle health management and operational efficiency.

#### 8.1.5. Machine Learning Approaches for Automated Monitoring of Behavioural Indicators Associated with Welfare: Brush Use in Dairy Cattle

Sadrzadeh Ahmadi [108] explored the use of machine learning to monitor natural scratching behaviour in dairy cattle through automated brush usage tracking. By integrating data on brush rotation with cow identity, captured either via low-frequency RFID or a fiducial marker-based computer vision system, the researchers developed a method to estimate daily brush use. The computer vision system outperformed the RFID system in terms of accuracy, and the application of machine learning further enhanced the precision of behavioural monitoring. This approach represents the first use of fiducial markers for automated group behaviour tracking in cattle and has the potential to serve as a welfare assessment tool and early illness detection method in farm management systems.

### 8.2. Applications of Sensing and Identification Technologies

Voogt, et al. [35] reviewed and outlined a variety of sensor technologies and artificial intelligence (AI) applications used to measure animal-based measures (ABMs) at the slaughterhouse. These include camera systems, such as high-speed imaging and computer vision, for detecting injuries, bruises, body condition, lameness, and organ abnormalities in broilers, pigs, and cattle. Microphones have been used in research to detect stress-related vocalizations, indicating pain or fear. Infrared sensors (e.g., ClassifEYE^®^, IRIS) assess issues like hock burns and wing injuries, while weight measurement systems (e.g., ChickSort 3.0, SmartWeigher, PigWei) track body condition and emaciation. Some commercially available systems, such as those by Meyn, Genba Solutions, and AI4Animals, integrate AI for automated inspection of carcasses, stunning responses, or bleeding rate. Despite promising developments, most systems are still in early research or limited commercial use, and only a few (like ChickenCheck and VisStick) have undergone external validation, highlighting the need for further research and regulatory approval before widespread implementation.

#### 8.2.1. RFID Tracking and Automated Weighing Systems in Practice

The primary goal in livestock production is efficient weight gain, reflected in average daily gains (ADGs) and average pen weights (APWs). While commercial farms typically weigh livestock only 2–3 times during growth, more frequent monitoring can help identify inefficiencies. Tools like Weight-Detect™ (by PLF Agritech Pty. Ltd., Toowoomba, Australia) or ref. [13] offer contactless monitoring, but adoption has been limited due to concerns about accuracy and technical issues [55]. Banhazi et al. [55] evaluated the precision of Weight-Detect across farms in Australia and Europe by comparing predicted APWs with manual scale measurements. The results showed predictive errors below 3%, supporting routine use in commercial settings, though accuracy may vary with factors like animal behaviour, camera placement, and farm practices.

The reported predictive error below 3% for the Weight-Detect system is encouraging, but several caveats apply. Banhazi et al. [55] note that accuracy varies with animal behaviour, camera placement, and farm-specific practices, variables that are difficult to control at scale and that introduce performance heterogeneity across deployment sites. Furthermore, the commercial evaluation was conducted across a limited number of farms in Australia and Europe, and the generalisability of these findings to diverse production systems, breeds, and feedlot layouts has not been established. A broader limitation of the automated weighing literature is the infrequent reporting of failure modes: published studies tend to report mean error under successful operation, with limited discussion of the proportion of weighing events excluded due to animal movement, crowding, or sensor malfunction. This selective reporting may lead to optimistic estimates of system reliability under full commercial conditions.

#### 8.2.2. Smart Troughs and Resource Utilization Monitoring Applications

As part of efforts to enhance environmental stewardship, many farmers are adopting practices that exclude cattle from streams and rivers, establishing riparian buffers to protect surface water quality. This shift necessitates the use of mechanical watering systems for livestock, which in turn introduces the need for reliable monitoring to detect system failures. In response to this challenge, a senior capstone project developed a solar-powered, wireless alert system to monitor water availability at remote troughs [82]. The system integrated three wireless communication technologies, Cellular, Wi-Fi, and Zigbee, to transmit water level data from multiple troughs to a central Raspberry Pi (RPi) hub located in the water pump house. Additional data, including water pressure and ambient temperature, were also monitored [82].

Each trough station was equipped with a solar-powered unit comprising an Arduino and an Xbee wireless module. The RPi aggregated data from all sources, storing it locally and issuing SMS alerts to the farmer if any parameter exceeded predefined thresholds. The project emphasized close collaboration with the client, who provided access to their farm for prototyping and field testing. This real-world deployment validated the system’s performance and reliability. The final report included a detailed cost analysis, projected savings, competitive benchmarking, and recommendations for future commercialization. Such technologies also underscore the potential of simple, modular, and IoT-based solutions [24] for improving water management in precision livestock systems.

#### 8.2.3. Location-Based Monitoring and Cattle Identification in Feedlot Operations

Cattle identification plays a central role in location-based monitoring systems essential for traceability, health tracking, and resource allocation. A systematic literature review of ML and DL approaches in vision-based identification showed that DL models outperform traditional ML in detection and robustness [12]. However, limitations in dataset quality and environmental control remain major barriers to implementation in real-world feedlots [87].

Key behavioural indicators such as an animal’s location, posture, and movement patterns play a crucial role in evaluating individual health and welfare status. A wide array of technologies now enables automated location tracking, including Global Navigation Satellite Systems (GNSS), Ultra-Wideband (UWB), Radio Frequency Identification (RFID), Wireless Sensor Networks (WSN) [106], and increasingly, computer vision systems. Each of these approaches offers unique strengths in terms of coverage, resolution, and cost, yet they also present limitations related to range, signal interference, and spatial accuracy.

Recent studies [106] suggest that integrating multiple sensing modalities, such as combining accelerometers with location-based technologies, through sensor fusion systems can enhance the granularity and reliability of behavioural insights. These multi-sensing frameworks enable a more comprehensive understanding of individual animal behaviour and provide a more robust basis for welfare assessment. Overall, location tracking technologies hold significant promise for advancing animal welfare monitoring in PLF systems. However, further interdisciplinary research is essential to overcome technical constraints and to optimize the deployment of such technologies under commercial farming conditions.

### 8.3. Systems for Monitoring Conditions and Behavioural Indicators That Influence Animal Health and Welfare

Non-intrusive animal health and welfare monitoring is essential because it minimizes stress and disturbance to livestock, ensuring natural behaviour and more accurate data collection [17]. This approach enhances animal well-being by avoiding physical restraints or invasive procedures, which can negatively impact health and productivity. Additionally, non-intrusive monitoring enables continuous, real-time data capture, allowing for timely detection of health issues and welfare concerns, ultimately improving management decisions and farm sustainability.

#### 8.3.1. Non-Intrusive and Non-Contact Health Monitoring Techniques in the Field

Image Analysis and Remote Diagnostic Sensing: Non-intrusive animal health and welfare monitoring can be effectively achieved through techniques based on image analysis and remote diagnostic sensing. Image analysis uses cameras and computer vision algorithms to observe and interpret animal behaviours, postures, and physical conditions without direct contact, enabling early detection of stress, lameness, or illness. Remote diagnostic sensing employs sensors that measure physiological parameters such as temperature, heart rate, or respiratory patterns from a distance, often using infrared or thermal imaging. Together, these technologies provide continuous, real-time insights while minimizing animal disturbance, enhancing welfare, and enabling proactive management.

#### 8.3.2. Welfare Assurance and Management Frameworks in Practice

Tuyttens et al. [21] critically examine the often-overlooked risks associated with Precision Livestock Farming (PLF) technologies, particularly concerning animal welfare. While PLF is widely promoted for its potential to enhance welfare outcomes, few welfare-focused technologies have been adopted in practice, and many claims remain unproven. The authors identify twelve potential threats to animal welfare, grouped into four categories: direct harm (e.g., technical failures, hardware discomfort), indirect harm via user behaviour (e.g., over-reliance, reduced human–animal interaction), harm through changes in housing and management, and ethical degradation (e.g., increased speciesism, further commodification of animals). The paper argues that while some direct risks can be mitigated through design and oversight, indirect and systemic risks are harder to anticipate or control. Ultimately, the paper calls for a balanced and cautious approach, where both the promises and perils of PLF are transparently assessed, to ensure technology truly serves animal welfare.

### 8.4. Regulatory Compliance and Technology Aided Ethical Considerations

Jongeneel, et al. [109] outlined interim findings from the project “Facilitating the CAP reform: Compliance and competitiveness of European agriculture”, focusing on the implementation of cross-compliance measures across seven EU member states, France, Germany, Italy, the Netherlands, the United Kingdom, Spain, and Poland. It included a comparative overview of how these countries have adopted the compliance framework, highlighting both the level of adherence by farmers and the associated costs of meeting regulatory requirements. In addition, the report extends its analysis to include similar policy implementations in three non-EU countries, Canada, the United States, and New Zealand, offering valuable international context for evaluating the effectiveness and economic impact of cross-compliance systems.

Animal-based measures (ABMs) are considered the most reliable indicators for assessing animal welfare; however, their manual evaluation during meat inspection is time-consuming and labour-intensive. Voogt et al. [35] explored the potential of sensor technology and artificial intelligence (AI) as tools for automating the scoring of ABMs, thereby improving efficiency and consistency. Drawing from existing review literature, this study provides an overview of ABMs currently recorded at slaughterhouses for poultry, pigs, and cattle, along with corresponding applications of sensor technologies. Additionally, relevant regulations and operational guidelines from the Dutch Regulatory Authority (RA) were examined to identify officially recognised ABMs. In total, sensor-based applications were reported for 10 of 37 ABMs identified in poultry, 13 of 41 in pigs, and 4 of 32 in cattle, with several technologies directly relating to meat inspection activities. Although European legislation mandates that official veterinarians conduct meat inspections, with limited exceptions for poultry post-mortem assessment, the findings highlight opportunities for the RA to incorporate sensor technologies as supportive tools to enhance inspection processes and deepen insight into animal welfare risks. A notable limitation, however, is the absence of external validation for many commercially available systems, which warrants further scrutiny and standardisation.

## 9. Opportunities and Implementation Challenges

Despite the significant potential of Precision Livestock Farming (PLF) technologies to enhance feedlot efficiency, animal welfare, and sustainability, their widespread adoption within feedlot operations remains constrained by several feedlot-specific barriers. These challenges extend beyond technical limitations and encompass economic, infrastructural, and socio-cultural dimensions that are unique to intensive cattle production systems.

For instance, Slayi et al. [35] investigated barriers to implementing climate-smart feedlot practices in rural areas of South Africa’s Eastern Cape. Their study highlighted several interrelated constraints relevant to feedlot adoption, including limited access to capital for infrastructure and technology investment, inadequate technical skills and knowledge among feedlot operators, unreliable resource availability such as water and feed, and prevailing cultural or social perceptions that influence attitudes towards intensive cattle production. Market accessibility and environmental concerns, such as land degradation or emissions management, were also identified as significant barriers.

Importantly, Slayi et al. [35] proposed targeted interventions to overcome these challenges, such as capacity-building programs to enhance technical expertise, financial support mechanisms to reduce investment barriers, and community engagement initiatives to address social and cultural reservations. These findings underscore the need for a holistic approach to technology adoption in feedlots, one that goes beyond technological readiness to address the practical, economic, and social realities faced by operators, particularly in resource-constrained or rural settings.

### 9.1. Opportunities

As outlined, the global livestock sector is undergoing a progressive digital transformation, with Precision Livestock Farming (PLF) technologies offering considerable potential to enhance productivity, promote animal welfare, and advance environmental sustainability [110,111]. Within smart feedlot systems, these technologies encompass a diverse suite of innovations, including RFID-based animal identification, automated feeding, weighing, and health-monitoring platforms [55,82]. Nonetheless, the widespread adoption of such technologies remains uneven, influenced by a complex interaction of technological, economic, regulatory, and organisational factors [39,40].

As we mentioned previously, feedlots are harsh environments, and the durability of most technologies is a significant barrier under such conditions. Dust, moisture, temperature extremes, and physical wear can quickly degrade equipment, making long-term reliability a critical concern.

PLF technologies introduce several compelling opportunities for modern livestock operations (e.g., [15]). First, they enhance operational efficiency by automating routine tasks such as feed delivery and weight monitoring, thereby reducing labour dependency and improving consistency in management practices [25,112]. Second, they enable data-driven decision-making, where integrated digital tools, such as farm accounting software, genetic databases, and carcass feedback systems, support optimized breeding, feeding, and marketing strategies [113]. This is particularly valuable in feedlot settings where small variations in management can significantly influence profitability.

Another significant opportunity lies in monitoring animal health and welfare. Technologies such as motion sensors, boluses, and automated dosing systems provide early alerts for health issues, facilitating timely interventions that can reduce mortality and antibiotic usage [114]. Additionally, PLF tools support environmental monitoring and compliance, with systems like NDVI sensors, precision irrigation controllers, and virtual fencing helping producers manage pastures more sustainably and comply with evolving environmental regulations.

Finally, PLF adoption can improve market access and product traceability. In high-value beef supply chains, data transparency and traceability are becoming prerequisites, especially for export markets. Technologies that support robust animal tracking and performance records thus offer a competitive advantage [26].

The integration of PLF technologies into smart feedlot systems offers substantial potential to transform livestock production, particularly in intensive operations where management precision is critical. Technologies such as automated feed delivery systems, RFID-based individual tracking, and real-time health monitoring using sensors enable highly granular control of animal growth, nutrition, and welfare. These systems improve feed conversion efficiency, reduce human error, and enhance traceability, which is increasingly important for meeting regulatory and consumer demands. Furthermore, the availability of decision-support platforms and carcass feedback systems allows feedlot operators to make data-driven adjustments that directly impact profitability and sustainability. As Behrendt et al. [113] and Becoña et al. [25] observed, tools related to feeding, weighing, and environmental monitoring are among the most adopted within PLF, and their functionality aligns closely with the operational needs of smart feedlots. Additionally, in the context of climate change and limited land resources, smart feedlots offer a controlled environment that supports climate-resilient livestock production through improved waste and water management [37]. These synergies position PLF as a foundational enabler for the evolution of conventional feedlots into more efficient, sustainable, and responsive production systems.

It should be noted, however, that the evidence base underpinning many of the claimed benefits of PLF technologies remains uneven. For several technology categories, including wearable biosensors, LLM-based decision support, and multi-sensor fusion platforms, peer-reviewed evidence of benefit is largely drawn from proof-of-concept or small-scale trial settings, and independent replication at commercial scale is limited. Reported outcomes such as reductions in antibiotic usage or improvements in feed conversion efficiency are frequently drawn from studies with short observation periods, single-site designs, or without appropriate control groups, making it difficult to attribute outcomes to the technology intervention with confidence. The absence of standardised reporting frameworks across the PLF literature, analogous to CONSORT for clinical trials or PRISMA for systematic reviews, compounds the difficulty of synthesising evidence and drawing reliable conclusions about effect sizes and generalisability.

### 9.2. Early Barriers to Adoption

Although several commercial technologies are available, particularly within the beef and dairy industries, widespread implementation is hindered by economic, infrastructural, and social barriers. High upfront costs, inadequate rural connectivity, and limited digital literacy among farm workers continue to restrict uptake, especially in developing regions. Furthermore, many PLF systems lack interoperability and user-friendly design, complicating integration with existing farm practices [37]. Overcoming these challenges will require targeted efforts, including reducing technology costs, investing in rural infrastructure, and providing tailored training to ensure practical, accessible adoption at scale.

Despite several advantages, widespread adoption of PLF technologies, especially in low- and middle-income regions, faces several early barriers. High upfront investment costs remain the most frequently cited constraint, particularly for small to medium-sized farms with limited access to capital [25,37]. Even when long-term benefits are demonstrable, the initial financial outlay can be prohibitive without targeted subsidies or financing models.

Awareness and skills gaps also hinder adoption. Some producers remain unaware of emerging technologies, or lack the digital literacy required for effective use and integration. This challenge is particularly acute in rural areas, where access to extension services and training is limited [37].

Infrastructure limitations, such as unreliable electricity and poor water access, present substantial barriers to smart feedlot development in developing regions. For instance, in the Eastern Cape of South Africa, inadequate infrastructure, knowledge gaps, and social resistance were shown to significantly inhibit the adoption of climate-smart feedlots [37]. These insights underscore the importance of aligning technological readiness with local infrastructure and cultural appropriateness to ensure effective uptake.

Further, producers often perceive a high risk of failure or low return on investment, especially in volatile production environments affected by drought, disease, or fluctuating market prices. Short-term constraints, such as urgent family needs or resource limitations, can further deprioritize technology investments [25]. Once again, the harsh conditions of feedlot environments play a significant role in producers’ distrust toward adopting new technologies.

Finally, sociocultural appropriateness influences adoption, as some technologies may be misaligned with the values, goals, or social norms of farming communities. Farmer motivations are diverse, and alignment with personal objectives, such as improving work-life balance or maintaining traditional methods, can significantly affect uptake [115].

### 9.3. Technical Challenges in System Integration

One of the primary hurdles in deploying PLF systems lies in the integration of heterogeneous hardware and software components. Sensors, cameras, RFID systems, and data processing units often originate from different manufacturers, leading to compatibility issues and communication bottlenecks. Ensuring seamless interoperability, synchronizing data streams, and maintaining real-time operation across platforms requires robust system architecture and ongoing technical support. Furthermore, integrating AI models into on-farm operations demands significant computational resources and careful tuning to align with dynamic field conditions.

### 9.4. Cost, Scalability, and Return on Investment

High initial costs of hardware installation, infrastructure upgrades, and system maintenance can deter small and medium-scale producers. While large operations may achieve economies of scale, scalability remains a concern in adapting these systems across diverse farm sizes and layouts. Additionally, demonstrating a clear return on investment (ROI) is critical for adoption; benefits such as improved productivity, reduced labour, or enhanced animal welfare must be quantified and communicated to justify expenditure. Without reliable financial benchmarks, many producers remain hesitant to invest.

### 9.5. Sustainable Agriculture and Environmental Pressures

The global push for sustainable agriculture is driven by the urgent need to balance food production with the protection of natural resources and ecological systems [38]. Livestock systems, particularly intensive operations like feedlots, face increasing scrutiny for their environmental impact, including greenhouse gas emissions, water consumption, nutrient runoff, and land degradation [45]. These pressures are amplified by climate change, biodiversity loss, and growing public demand for transparent and environmentally responsible food systems.

Sustainability in this context requires more than just input efficiency; it demands a system-level approach that integrates ecological, technological, and social dimensions [54]. For example, methane emissions from cattle are a major contributor to agricultural greenhouse gases [45], while poor manure management can lead to nutrient leaching and waterway contamination [73,74]. Addressing these challenges requires locally adapted solutions that improve resource efficiency and reduce waste without compromising productivity.

Smart feedlot systems, enabled by Precision Livestock Farming (PLF), offer powerful tools to advance sustainability goals. Automated precision feeding technologies reduce overfeeding and feed waste, thereby lowering enteric methane emissions per unit of beef produced [82]. IoT-enabled water monitoring systems help detect leaks and optimize consumption, while real-time environmental sensors track humidity, ammonia, and temperature, ensuring optimal housing conditions and reducing the risk of disease outbreaks and pollution.

Frameworks such as socioecological models [54] are valuable for assessing the trade-offs between productivity, welfare, and environmental outcomes. These tools guide decision-making at the intersection of technological innovation and ecological stewardship, supporting the design of resilient livestock systems that align with long-term sustainability targets. In Australia, where livestock is a significant source of emissions and water stress is an ongoing concern, such models are particularly relevant for balancing industry growth with environmental responsibility [45].

As climate and regulatory pressures intensify, sustainability will not be optional for feedlot operators; it will be a defining constraint and opportunity. Smart systems that can deliver both efficiency and environmental accountability will play a central role in shaping the future of livestock production.

### 9.6. Training, Acceptance, and Human Factors

Adoption of PLF technologies also depends heavily on user acceptance and capacity building. Many farm operators and workers lack the technical background to operate, troubleshoot, or interpret data from advanced systems. Resistance to change, perceived complexity, and fears of technological redundancy can limit user engagement. Therefore, training programs, user-friendly interfaces, and participatory design approaches are essential to build confidence and ensure that the technologies align with daily workflows and practical needs.

A critical but often underexplored dimension of technology readiness concerns the specific skills, knowledge, and computational infrastructure required of farm operators. Current evidence suggests that large commercial feedlot operations with dedicated technical staff are increasingly positioned to meet these demands, while small to medium-scale producers face substantial readiness gaps related to digital literacy, connectivity, and maintenance capacity [40,112]. Targeted capacity-building programs, simplified user interfaces, and vendor-supported onboarding pathways have been identified as practical interventions to bridge this gap [37]. Without deliberate investment in workforce capability alongside technological infrastructure, the modernisation benefits of smart feedlot systems will remain inaccessible to a significant portion of the global beef production sector.

### 9.7. Connectivity Constraints in Rural Environments

Effective PLF systems rely on stable data transmission and cloud connectivity, yet rural and remote farming areas often suffer from poor internet infrastructure. Limited bandwidth, intermittent connectivity, and high latency hinder real-time data collection, cloud-based analytics, and remote support. While edge computing and local storage solutions can alleviate some constraints, persistent connectivity issues remain a barrier to the full deployment of IoT-enabled livestock monitoring systems. Addressing these infrastructure gaps is vital for equitable access to smart farming solutions across geographic regions.

## 10. Future Directions: Intelligent and Autonomous Feedlots

The modernisation of feedlot operations through intelligent and autonomous systems represents both the logical trajectory of the technological advances reviewed in this paper and a significant practical challenge requiring coordinated progress across technology development, infrastructure investment, workforce capability, and governance frameworks.

Our current experiences with using AI in food production suggest that, in the not-too-distant future, intelligent ecosystems will emerge where data, machines, and decision-making converge seamlessly. We hypothesize that in future smart feedlot systems, livestock will be continuously monitored through a dense network of biosensors, drones will autonomously manage herd inspections, and smart feeders will deliver precisely tailored nutrition in real time. Decision-making will be powered by large language models (LLMs) and retrieval-augmented generation (RAG) systems that synthesize global research knowledge with local farm data, enabling proactive interventions [116,117]. These fully integrated systems will not only optimize productivity and animal welfare but also uphold sustainability standards and ethical practices, creating resilient agricultural systems equipped to meet future challenges. We would expect future feedlots to evolve toward a more integrated model that blends biological systems with machine intelligence, potentially setting a new direction for livestock management in the 21st century.

### 10.1. Fully Autonomous Feedlot Management

Based on our direct experience developing AI technologies for feedlot operations, we observe that the trajectory of feedlot management is decisively moving toward fully autonomous systems, where human oversight is minimized and adaptive intelligence becomes central to daily operations. In our work, we have seen how autonomous systems can be deployed to assist with managing tasks such as controlled feeding and real-time health monitoring. At the heart of this transformation there will be automation and decision support systems powered by AI and data mining techniques, which extract actionable patterns from large, dynamic datasets encompassing animal behaviour and environmental conditions. By leveraging continuous streams of visual data, these systems enable predictive interventions that improve both animal welfare and resource efficiency.

Looking ahead, we believe that Large Language Models (LLMs) and Retrieval-Augmented Generation (RAG) frameworks hold immense promise. These tools can synthesize domain knowledge from literature and farm-specific data in real time, guiding autonomous decision-making, troubleshooting, and operational strategy with minimal human input.

### 10.2. Emerging Technologies in AI, Robotics, and Sensing

Building on our firsthand insights, we also recognize that the broader landscape of technological innovation is rapidly evolving. Emerging technologies are redefining the capabilities of AI, robotics, and sensing in feedlot environments. Machine vision systems employing deep convolutional neural networks are reaching unprecedented accuracies in detecting early signs of lameness, respiratory distress, or behavioural anomalies. Robotics is shifting toward more mobile and context-aware systems, combining sensor fusion and reinforcement learning to navigate the complex physical environments of feedlots. Simultaneously, the next wave of biosensors, wearable, implantable, and environmental biosensors will produce massive real-time datasets. Here, data mining becomes critical to identifying subtle, high-value insights buried in high-frequency signals. On top of this, LLMs fine-tuned on animal science and veterinary datasets, coupled with RAG pipelines, could act as intelligent agents, provide on-the-fly interpretation of sensor data, and generate adaptive management recommendations. These AI-driven systems are poised to function as intelligent intermediaries, offering scalable and continuously learning support for autonomous decision systems in feedlot operations.

### 10.3. Policy, Data Governance, and Interoperability Standards

As the digitization of feedlots accelerates, the governance of data becomes both more complex and more critical. Future policy frameworks must not only address data ownership, privacy, and ethical deployment of AI but also enable transparent and explainable decision-making processes, especially when LLMs and RAG systems are embedded in core operational workflows. Standardising interoperability protocols will be vital to ensure that sensors, robotics, and data platforms can cohesively interact in a diverse, competitive marketplace. Open, federated data architectures could facilitate safe and effective data mining across multi-farm datasets, leading to industry-wide improvements in disease management, welfare assessment, and sustainability metrics. Research opportunities are abundant in creating hybrid governance models that balance competitive innovation with shared intelligence, while simultaneously ensuring the safe and equitable deployment of advanced AI systems in global feedlot industries.

## 11. Conclusions

Smart feedlot technologies are fundamentally transforming the livestock industry, enabling unprecedented levels of precision, efficiency, and sustainability across the full spectrum of feedlot operations. This review has synthesised evidence from approximately 350 publications spanning digital infrastructure, animal identification and sensing, machine vision, AI-based analytics, and decision support systems, of which 117 are formally cited as representative references; the remainder informed the thematic synthesis without individual citation. Across these thematic areas, a consistent picture emerges: key components have progressed beyond proof-of-concept toward operation under commercial constraints, yet significant gaps remain between demonstrated capability and widespread adoption. Among the technologies reviewed, vision-based monitoring systems, particularly those employing deep learning architectures such as YOLO-based models, have shown the most rapid maturation, achieving robust performance in cattle identification, behavioural profiling, lameness detection, and liveweight estimation under real-world conditions. RFID-based tracking and automated weighing systems have demonstrated predictive errors below 3% in representative commercial deployments, supporting their integration into routine feedlot management. Sensor fusion approaches combining visual, thermal, and non-visual modalities have shown particular promise in addressing persistent challenges such as occlusion, environmental variability, and individual animal re-identification. The emergence of LLM and RAG-based decision support frameworks represents a significant frontier, offering the potential to bridge the gap between raw sensor data and actionable, natural-language farm management recommendations. Despite these advances, several critical research gaps need urgent attention. In the domain of computer vision and AI, while detection accuracy under controlled conditions is well documented, performance under the full range of feedlot environmental conditions, including dust, variable lighting, extreme weather, and high animal density, remains insufficiently characterised. Occlusion handling in densely populated pens has not been systematically addressed in feedlot-specific studies, and most re-identification frameworks have been validated on small, homogeneous datasets that do not reflect the diversity of commercial cattle populations. In the domain of sensing and infrastructure, rumen bolus technologies show promise for early disease detection, yet sensitivity and specificity data under commercial feedlot conditions remain limited, and cost-benefit analyses are largely absent from the literature. Wireless network architectures for large-scale feedlot deployments, particularly under intermittent connectivity and high device density, have received limited empirical evaluation. In the domain of decision support and AI analytics, LLM and RAG-based systems for livestock management are in their infancy, with no published large-scale validation of their deployment in operational feedlot environments. The integration of these systems with heterogeneous sensor platforms and existing farm management software remains an open engineering challenge. In the domain of adoption and implementation, the evidence base for return on investment across diverse farm sizes, production systems, and geographic contexts is thin, and sociotechnical barriers to adoption, including digital literacy, cultural appropriateness, and workforce capability, remain underexplored in the peer-reviewed literature. The absence of standardised performance benchmarking across studies makes it difficult to compare systems or establish industry-wide deployment thresholds. Interoperability between heterogeneous sensor platforms, data management systems, and decision support tools remains a major barrier, with most deployments remaining siloed rather than integrated. Furthermore, the durability and maintenance burden of precision technologies in harsh feedlot conditions continues to constrain long-term reliability and producer confidence. Looking ahead, we identify four priority directions for future research and development. First, the field urgently requires longitudinal, commercial-scale validation studies that assess not only technical performance but also economic outcomes, welfare impacts, and return on investment across diverse farm sizes, breeds, and production systems. Second, the development of cost-effective, ruggedised sensing solutions specifically engineered for feedlot environments, rather than adapted from other domains, should be a central focus of applied research. Third, the integration of LLM and RAG frameworks with structured feedlot databases and real-time sensor streams offers a compelling pathway toward genuinely autonomous decision support, and represents one of the most promising near-term research opportunities in precision livestock farming. Fourth, the establishment of governance frameworks for federated livestock data, addressing data ownership, privacy, interoperability standards, and equitable access, is essential to enable industry-wide learning from multi-farm datasets while protecting producer interests. Continued interdisciplinary collaboration between engineers, animal scientists, veterinarians, data scientists, and industry partners will be essential to realise the vision of resilient, scalable, and welfare-centric feedlot operations. Smart feedlots are not merely a technological aspiration; they represent a necessary evolution in livestock production, one capable of simultaneously meeting the demands of productivity, animal welfare, environmental accountability, and food system transparency in the 21st century.

## Figures and Tables

**Table 1:** Boolean search strings applied across databases in the systematic literature search.

Database	Boolean Search String
Web of Science & Scopus—String 1 *Core technology and system scope*	(“smart feedlot” OR “precision livestock farming” OR “PLF”) AND (“beef cattle” OR “feedlot cattle”) AND (“sensor” OR “RFID” OR “computer vision” OR “machine vision” OR “artificial intelligence” OR “machine learning” OR “deep learning” OR “IoT” OR “Internet of Things”)
Web of Science & Scopus—String 2 *Animal health and welfare monitoring*	(“feedlot” OR “beef cattle”) AND (“health monitoring” OR “welfare monitoring” OR “behaviour monitoring” OR “disease detection” OR “lameness” OR “bovine respiratory disease”) AND (“automated” OR “sensor” OR “camera” OR “wearable” OR “image analysis”)
Web of Science & Scopus—String 3 *Infrastructure and data systems*	(“feedlot” OR “livestock”) AND (“wireless network” OR “edge computing” OR “cloud computing” OR “data analytics” OR “decision support” OR “digital twin” OR “LLM” OR “large language model” OR “retrieval-augmented generation” OR “RAG”)
Web of Science & Scopus—String 4 *Weight estimation and feeding*	(“beef cattle” OR “feedlot”) AND (“liveweight estimation” OR “automated weighing” OR “feed intake monitoring” OR “bunk management” OR “precision feeding”) AND (“image” OR “RFID” OR “sensor” OR “algorithm”)
Google Scholar & specialist journals *Frontiers in Veterinary Science;* *Computers and Electronics in Agriculture; Animals*	Adapted keyword combinations from Strings 1–4, supplemented by: “stockperson behaviour”, “human-animal interaction feedlot”, “UAV livestock monitoring”, “rumen bolus”, “accelerometer cattle”, “thermal imaging livestock”, “occlusion cattle tracking”, “GIS feedlot management”
**Time range**	2015–2025 (primary); foundational references from ∼2000 where contextually essential
**Filters**	English language; peer-reviewed journals, conference proceedings, and recognised technical reports; full text available; PhD theses; opinion pieces excluded

**Table 2:** Approximate number of reviewed publications by thematic area. (*) Key references are illustrative examples only.

Thematic Area	No. of Pubs.	Key References *
Smart Feedlot Concepts and Definitions	40–50	[4,17,18,20,21]
PLF and Digital Agriculture	60–70	[18,20,21,22,23]
Animal Welfare and Human-Animal Interaction	50–60	[19,29,30,31,32]
Machine Vision, Sensors, and Infrastructure	70–80	[24,25,27,28,33]
Behaviour Monitoring and AI Technologies	50–60	[6,8,11,34]
Post-Feedlot Operations and Welfare at Slaughter	20–30	[9,17,35,36]
Adoption, Challenges, and Sustainability	40–50	[21,37,38,39,40]
Environmental Management and Resource Use	30–40	[41,42,43,44,45]
**Total**	**350–400**	

**Table 3:** Key differences between feedlots and paddocks.

Feature	Feedlot	Paddock
Feeding System	Grain-based diet	Grass-based grazing
Space	Confined area	Open pasture
Purpose	Rapid weight gain	Natural grazing
Manure Management	Needs waste removal	Naturally fertilizes soil
Environmental Impact	Higher due to waste concentration	Lower with proper grazing
Stocking Density	High (many cattle in a small area)	Low (fewer cattle per acre)
Best For	Finishing cattle for beef production	Sustainable livestock farming

**Table 4:** Single-hop vs. multi-hop connections.

Feature	Single-Hop	Multi-Hop
Communication	Direct (point-to-point)	Uses multiple relay nodes
Range	Limited to device power	Can cover large distances
Reliability	A single failure can break communication	Alternative paths ensure data reaches its destination
Energy Use	Requires strong signals	More energy-efficient

## Data Availability

No new data were created or analyzed in this study. Data sharing is not applicable to this article.

## References

[1] Fortune Business Insights (2024). Beef Market Size, Share & Industry Analysis, By Cut Type (Ground, Roasts, Steaks, and Others), and Distribution Channel (Retail Sales, HoReCa, and Butcher Shops), and Regional Forecast, 2025–2032.

[2] Wongpiyabovorn O., Wang T., Menendez H., Yago A.L. (2025). Precision livestock farming technologies in beef cattle production: Current and future. Choices.

[3] Losacco C., Pugliese G., Forte L., Tufarelli V., Maggiolino A., De Palo P. (2025). Digital Transition as a Driver for Sustainable Tailor-Made Farm Management: An Up-to-Date Overview on Precision Livestock Farming. Agriculture.

[4] Wagner J.J., Archibeque S.L., Feuz D.M. (2014). The modern feedlot for finishing cattle. Annu. Rev. Anim. Biosci..

[5] Johnstone S. (2024). An Economic Feasibility Study of a Managed Supply Chain Stocker Operation in NE Oklahoma. Master’s Thesis.

[6] Anagnostopoulos A., Griffiths B.E., Siachos N., Neary J., Smith R.F., Oikonomou G. (2023). Initial validation of an intelligent video surveillance system for automatic detection of dairy cattle lameness. Front. Vet. Sci..

[7] Kurras F., Jakob M. (2024). Smart dairy farming—The potential of the automatic monitoring of dairy cows’ behaviour using a 360-degree camera. Animals.

[8] Guarnido-Lopez P., Ramirez-Agudelo J.F., Denimal E., Benaouda M. (2024). Programming and setting up the object detection algorithm YOLO to determine feeding activities of beef cattle: A comparison between YOLOv8m and YOLOv10m. Animals.

[9] Delmore R.J. (2022). Automation in the global meat industry. Encyclopedia of Meat Sciences.

[10] Barbut S. (2014). Automation and meat quality-global challenges. Meat Sci..

[11] Mu Y., Hu J., Wang H., Li S., Zhu H., Luo L., Wei J., Ni L., Chao H., Hu T. (2024). Research on the behavior recognition of beef cattle based on the improved lightweight CBR-YOLO model based on YOLOv8 in multi-scene weather. Animals.

[12] Li Z., Zhang Y., Kang X., Mao T., Li Y., Liu G. (2025). Individual Recognition of a Group Beef Cattle Based on Improved YOLO v5. Agriculture.

[13] Lan L., Shen L., Wang H., Yao Y., Zheng P., Mao A. (2024). Estimation of cattle weight from composite image/height/length data with spatial and channel attention convolution network (SCA-ConvNet). Signal Image Video Process..

[14] Luke M.M.E., Burgess J.E.M., Gonzalez L.A. (2024). Breed differences in feeding behavior measured by two remote monitoring technologies in feedlot steers. J. Anim. Sci..

[15] Platts S.V., McMeniman J.P., Lees A.M., George M.H., George M.M., Gaughan J.B. (2025). Effect of algorithm-based feed allocation on performance, health, and carcass outcomes of Brahman cross steers. Smart Agric. Technol..

[16] Qiu H., Wang X., Shen J., Yang S., Zhao W. (2025). Building a Cattle Farming System in Industry 4.0. Proceedings of the International Conference on Blockchain and Trustworthy Systems.

[17] Tzanidakis C., Tzamaloukas O., Simitzis P., Panagakis P. (2023). Precision Livestock Farming Applications (PLF) for Grazing Animals. Agriculture.

[18] Morrone S., Dimauro C., Gambella F., Cappai M.G. (2022). Industry 4.0 and Precision Livestock Farming (PLF): An up to Date Overview across Animal Productions. Sensors.

[19] Schillings J., Bennett R., Rose D.C. (2021). Exploring the potential of precision livestock farming technologies to help address farm animal welfare. Front. Anim. Sci..

[20] Norton T., Chen C., Larsen M.L.V., Berckmans D. (2019). Review: Precision livestock farming: Building ‘digital representations’ to bring the animals closer to the farmer. Animal.

[21] Tuyttens F.A., Molento C.F., Benaissa S. (2022). Twelve threats of precision livestock farming (PLF) for animal welfare. Front. Vet. Sci..

[22] Trapanese L., Bifulco G., Macchio A.C., Aragona F., Purrone S., Campanile G., Salzano A. (2025). Precision Livestock Farming applied to the dairy sector: 50 years of history with a text mining and topic analysis approach. Smart Agric. Technol..

[23] Marino R., Petrera F., Abeni F. (2023). Scientific productions on precision livestock farming: An overview of the evolution and current state of research based on a bibliometric analysis. Animals.

[24] Michie C., Andonovic I., Davison C., Hamilton A., Tachtatzis C., Jonsson N., Duthie C.A., Bowen J., Gilroy M. (2020). The Internet of Things enhancing animal welfare and farm operational efficiency. J. Dairy Res..

[25] Becoña J.P., Grané M., Miguez M., Arnaud A. (2024). LoRa, Sigfox, and NB-IoT: An empirical comparison for IoT LPWAN technologies in the agribusiness. IEEE Embed. Syst. Lett..

[26] Wolfert S., Ge L., Verdouw C., Bogaardt M.-J. (2017). Big data in smart farming—A review. Agric. Syst..

[27] Wurtz K., Camerlink I., D’Eath R.B., Fernández A.P., Norton T., Steibel J., Siegford J. (2019). Recording behaviour of indoor-housed farm animals automatically using machine vision technology: A systematic review. PLoS ONE.

[28] Astill J., Dara R.A., Fraser E.D.G., Roberts B., Sharif S. (2020). Smart poultry management: Smart sensors, big data, and the internet of things. Comput. Electron. Agric..

[29] Rushen J., de Passillé A.M. (2008). Stockmanship and the Interactions between People and Cattle. The Welfare of Cattle.

[30] Hemsworth P.H., Barnett J.L., Coleman G.J. (2009). The integration of human-animal relations into animal welfare monitoring schemes. Anim. Welf..

[31] Salvin H.E., Lees A.M., Cafe L.M., Colditz I.G., Lee C. (2020). Welfare of beef cattle in Australian feedlots: A review of the risks and measures. Anim. Prod. Sci..

[32] Buller H., Blokhuis H., Lokhorst K., Silberberg M., Veissier I. (2020). Animal Welfare Management in a Digital World. Animals.

[33] Yamamoto Y., Akizawa K., Aou S., Taniguchi Y. (2025). Entire-barn dairy cow tracking framework for multi-camera systems. Comput. Electron. Agric..

[34] Dominiak K.N., Kristensen A.R. (2017). Prioritizing alarms from sensor-based detection models in livestock production—A review on model performance and alarm reducing methods. Comput. Electron. Agric..

[35] Voogt A.M., Schrijver R.S., Temürhan M., Bongers J.H., Sijm D.T.H.M. (2023). Opportunities for Regulatory Authorities to Assess Animal-Based Measures at the Slaughterhouse Using Sensor Technology and Artificial Intelligence: A Review. Animals.

[36] Edwards-Callaway L., Loh H.Y., Kautsky C., Sullivan P. (2025). A Comparison of Artificial Intelligence and Human Observation in the Assessment of Cattle Handling and Slaughter. Animals.

[37] Slayi M., Zhou L., Jaja I.F. (2023). Constraints Inhibiting Farmers’ Adoption of Cattle Feedlots as a Climate-Smart Practice in Rural Communities of the Eastern Cape, South Africa: An In-Depth Examination. Sustainability.

[38] Velten S., Leventon J., Jager N., Newig J. (2015). What is sustainable agriculture? A systematic review. Sustainability.

[39] Dill M.D., Emvalomatis G., Saatkamp H., Rossi J.A., Pereira G.R., Barcellos J.O.J. (2015). Factors affecting adoption of economic management practices in beef cattle production in Rio Grande do Sul state, Brazil. J. Rural Stud..

[40] Makinde A., Islam M.M., Wood K.M., Conlin E., Williams M., Scott S.D. (2022). Investigating perceptions, adoption, and use of digital technologies in the Canadian beef industry. Comput. Electron. Agric..

[41] Menendez H., Tedeschi L. (2020). The characterization of the cow-calf, stocker and feedlot cattle industry water footprint to assess the impact of livestock water use sustainability. J. Agric. Sci..

[42] Cusack D.F., Kazanski C.E., Hedgpeth A., Chow K., Cordeiro A.L., Karpman J., Ryals R. (2021). Reducing climate impacts of beef production: A synthesis of life cycle assessments across management systems and global regions. Glob. Change Biol..

[43] Palangi V., Lackner M. (2022). Management of enteric methane emissions in ruminants using feed additives: A review. Animals.

[44] Werth S.J., Rocha A.S., Oltjen J.W., Kebreab E., Mitloehner F.M. (2021). A life cycle assessment of the environmental impacts of cattle feedlot finishing rations. Int. J. Life Cycle Assess..

[45] Muir S.K. (2011). Greenhouse Gas Emissions from Australian Beef Feedlots. Ph.D. Thesis.

[46] Parlato M.C., Valenti F., Porto S.M. (2024). GIS-based methodology for tracking the grazing cattle site use. Heliyon.

[47] McFadden J., Erickson B., Lowenberg-DeBoer J., Milics G. Global adoption of precision agriculture: An update on trends and emerging technologies. Proceedings of the 16th International Conference on Precision Agriculture.

[48] Felius M., Beerling M.-L., Buchanan D.S., Theunissen B., Koolmees P.A., Lenstra J.A. (2014). On the history of cattle genetic resources. Diversity.

[49] Pacyga D.A. (2020). Slaughterhouse: Chicago’s Union Stock Yard and the World It Made.

[50] Specht J. (2019). Red Meat Republic: A Hoof-to-Table History of How Beef Changed America.

[51] Young J. (2018). Continental European Beef Breeds: Their Use and Impact on the United States Beef Industry. Ph.D. Thesis.

[52] Frazier E., Sprott L., Sanders J., Dahm P., Crouch J., Turner J. (1999). Sire marbling score expected progeny difference and weaning weight maternal expected progeny difference associations with age at first calving and calving interval in Angus beef cattle. J. Anim. Sci..

[53] Reinhardt C., Busby W., Corah L. (2009). Relationship of various incoming cattle traits with feedlot performance and carcass traits. J. Anim. Sci..

[54] Robertson G.P. (2015). A sustainable agriculture?. Daedalus.

[55] Banhazi T., Dunn M., Banhazi A. (2022). Weight-Detect™: On-farm evaluation of the precision of image analysis based weight prediction system. Practical Precision Livestock Farming.

[56] Richeson J.T., Lawrence T.E., White B.J. (2018). Using advanced technologies to quantify beef cattle behavior. Transl. Anim. Sci..

[57] Edwards T. (2010). Control methods for bovine respiratory disease for feedlot cattle. Vet. Clin. Food Anim. Pract..

[58] He Z.X., He M.L., Zhao Y.L., Xu L., Walker N.D., Beauchemin K.A., McAllister T.A., Yang W.Z. (2015). Effect of wheat dried distillers grains and enzyme supplementation on growth rates, feed conversion ratio and beef fatty acid profile in feedlot steers. Animal.

[59] Henchion M., McCarthy M., Resconi V.C., Troy D. (2014). Meat consumption: Trends and quality matters. Meat Sci..

[60] Delgado C.L. (2003). Rising Consumption of Meat and Milk in Developing Countries Has Created a New Food Revolution. J. Nutr..

[61] González A.D., Frostell B., Carlsson-Kanyama A. (2011). Protein efficiency per unit energy and per unit greenhouse gas emissions: Potential contribution of diet choices to climate change mitigation. Food Policy.

[62] Food and Agriculture Organization (2022). The State of the World’s Land and Water Resources for Food and Agriculture 2021: Systems at Breaking Point.

[63] Samuelson K., Hubbert M., Galyean M., Löest C. (2016). Nutritional recommendations of feedlot consulting nutritionists: The 2015 New Mexico State and Texas Tech University survey. J. Anim. Sci..

[64] Vasconcelos J., Galyean M. (2007). Nutritional recommendations of feedlot consulting nutritionists: The 2007 Texas Tech University survey. J. Anim. Sci..

[65] González L., Kyriazakis I., Tedeschi L. (2018). Precision nutrition of ruminants: Approaches, challenges and potential gains. Animal.

[66] Herd R., Archer J., Arthur P. (2003). Reducing the cost of beef production through genetic improvement in residual feed intake: Opportunity and challenges to application. J. Anim. Sci..

[67] Robinson D.L., Oddy V. (2004). Genetic parameters for feed efficiency, fatness, muscle area and feeding behaviour of feedlot finished beef cattle. Livest. Prod. Sci..

[68] Mueller M.L., Van Eenennaam A.L. (2022). Synergistic power of genomic selection, assisted reproductive technologies, and gene editing to drive genetic improvement of cattle. CABI Agric. Biosci..

[69] Seo D., Lee D.H., Jin S., Won J.I., Lim D., Park M., Kim T.H., Lee H.K., Kim S., Choi I. (2022). Long-term artificial selection of Hanwoo (Korean) cattle left genetic signatures for the breeding traits and has altered the genomic structure. Sci. Rep..

[70] Lee T.L., Terrell S.P., Bartle S.J., Apley M.D., Rethorst D., Thomson D.U., Reinhardt C.D. (2015). Current feedlot cattle health and well-being program recommendations in the United States and Canada: The 2014 feedlot veterinary consultant survey. Bov. Pract..

[71] Marcillac-Embertson N., Robinson P., Fadel J., Mitloehner F. (2009). Effects of shade and sprinklers on performance, behavior, physiology, and the environment of heifers. J. Dairy Sci..

[72] Dahlin A., Emanuelsson U., McAdam J. (2005). Nutrient management in low input grazing-based systems of meat production. Soil Use Manag..

[73] Wang Y., Ghimire S., Wang J., Dong R., Li Q. (2021). Alternative management systems of beef cattle manure for reducing nitrogen loadings: A case-study approach. Animals.

[74] Agga G.E., Couch M., Parekh R.R., Mahmoudi F., Appala K., Kasumba J., Loughrin J.H., Conte E.D. (2022). Lagoon, anaerobic digestion, and composting of animal manure treatments impact on tetracycline resistance genes. Antibiotics.

[75] Jensen P.D., Sullivan T., Carney C., Batstone D.J. (2014). Analysis of the potential to recover energy and nutrient resources from cattle slaughterhouses in Australia by employing anaerobic digestion. Appl. Energy.

[76] Dotts H.A. (2025). Optimizing Heifer Development on Dormant Rangelands by Integrating Precision Livestock Technologies with the Range Supplementation Model and the Evaluation of Short-Term Rotations of Precision Technology on Plant Communities. Ph.D. Thesis.

[77] Hemsworth P.H., Coleman G.J. (2011). Human-Livestock Interactions: The Stockperson and the Productivity and Welfare of Intensively Farmed Animals.

[78] Destrez A., Haslin E., Boivin X. (2018). What stockperson behavior during weighing reveals about the relationship between humans and suckling beef cattle: A preliminary study. Appl. Anim. Behav. Sci..

[79] Neethirajan S. (2024). Artificial Intelligence and Sensor Innovations: Enhancing Livestock Welfare with a Human-Centric Approach. Hum.-Centric Intell. Syst..

[80] Mintline E.M., Stewart M., Rogers A.R., Cox N.R., Verkerk G.A., Stookey J.M., Webster J.R., Tucker C.B. (2013). Play behavior as an indicator of animal welfare: Disbudding in dairy calves. Appl. Anim. Behav. Sci..

[81] Croney C., Apley M., Capper J., Mench J., Priest S. (2012). Bioethics symposium: The ethical food movement: What does it mean for the role of science and scientists in current debates about animal agriculture?. J. Anim. Sci..

[82] Salib E. A cost effective smart trough monitoring alert system. Proceedings of the 2022 ASEE Annual Conference & Exposition.

[83] Alanezi M.A., Shahriar M.S., Hasan M.B., Ahmed S., Sha’aban Y.A., Bouchekara H.R. (2022). Livestock management with unmanned aerial vehicles: A review. IEEE Access.

[84] Czurkó D., Fehér G. (2023). AI-Assisted Drone Localization of Arbitrary Objects using Aruco Markers. 1st Workshop on Intelligent Infocommunication Networks, Systems and Services (WI2NS2).

[85] Longmore S.N., Collins R.P., Pfeifer S., Fox S.E., Mulero-Pázmány M., Bezombes F., Goodwin A., De Juan Ovelar M., Knapen J.H., Wich S.A. (2017). Adapting astronomical source detection software to help detect animals in thermal images obtained by unmanned aerial systems. Int. J. Remote Sens..

[86] Milan H.F.M., Perano K.M., Gebremedhin K.G. Survey and future prospects in precision dairy farming. Proceedings of the 10th International Livestock Environment Symposium (ILES X).

[87] Hossain M.E., Kabir M.A., Zheng L., Swain D.L., McGrath S., Medway J. (2022). A systematic review of machine learning techniques for cattle identification: Datasets, methods and future directions. Artif. Intell. Agric..

[88] Flores H., Meneses C., Villalobos J.R., Sanchez O. (2017). Improvement of feedlot operations through statistical learning and business analytics tools. Comput. Electron. Agric..

[89] Balaguer A., Benara V., Cunha R.L., Hendry T., Holstein D., Marsman J., Mecklenburg N., Malvar S., Nunes L.O., Padilha R. (2024). RAG vs fine-tuning: Pipelines, tradeoffs, and a case study on agriculture. arXiv.

[90] Siachos N., Neary J.M., Smith R.F., Oikonomou G. (2024). Automated dairy cattle lameness detection utilizing the power of artificial intelligence; current status quo and future research opportunities. Vet. J..

[91] Chuah W.Q. (2022). Towards Building a Vet-Assist System: Animal pose estimation and counting walking steps. Australasian Conference on Robotics and Automation 2022.

[92] Li N., Ren Z., Li D., Zeng L. (2020). Review: Automated techniques for monitoring the behaviour and welfare of broilers and laying hens: Towards the goal of precision livestock farming. Animal.

[93] Claire S. (2024). RFID and Camera Technology Beef up Cattle Management. RFID Journal.

[94] Kidane N.F., Irvin M.E., Foxworth W.B., Carstens G.E., Horner S., O’Reilly K. (2023). 377 Evaluation of Feed Intake, Feed Efficiency and Days on Trial in Growing Goats Fed a Total Mixed Diets Using Growsafe Feeding Technology. J. Anim. Sci..

[95] Dickson E.C., Kayser W.C., Latham C.M., Leatherwood J.L., Daigle C.L., White S.H. (2019). Evaluating equine feeding behavior utilizing GrowSafe Systems: A pilot study. Transl. Anim. Sci..

[96] La C.-A., Varga L.-O., Heusse M., Duda A. (2014). Energy-efficient multi-hop broadcasting in low power and lossy networks. Proceedings of the 17th ACM International Conference on Modeling, Analysis and Simulation of Wireless and Mobile Systems.

[97] (2006). IEEE Standard for Information Technology—Local and Metropolitan Area Networks—Specific Requirements—Part 15.4: Wireless Medium Access Control (MAC) and Physical Layer (PHY) Specifications for Low Rate Wireless Personal Area Networks (WPANs).

[98] Jacobs J.L., Hersom M.J., Andrae J.G., Duckett S.K. (2023). Training and adaptation of beef calves to precision supplementation technology for individual supplementation in grazing systems. Animals.

[99] Wang Y., Mücher S., Wang W., Guo L., Kooistra L. (2023). A review of three-dimensional computer vision used in precision livestock farming for cattle growth management. Comput. Electron. Agric..

[100] Chen X., Davis J. (2000). Camera Placement Considering Occlusion for Robust Motion Capture.

[101] Rahimian P., Kearney J.K. (2016). Optimal camera placement for motion capture systems. IEEE Trans. Vis. Comput. Graph..

[102] Alempijevic A., Vidal-Calleja T., Falque R., Walmsley B., McPhee M. (2025). 3D imaging for on-farm estimation of live cattle traits and carcass weight prediction. Meat Sci..

[103] Song Z., Zhang J., Wu Z., Du Y., Hu W., Liu X. (2023). Methods of three-dimensional reconstruction and body size measurement of cattle based on multi-view binocular camera. Third International Conference on Signal Image Processing and Communication (ICSIPC 2023).

[104] Transue S., Nguyen P., Vu T., Choi M.-H. (2017). Thermal-depth fusion for occluded body skeletal posture estimation. 2017 IEEE/ACM International Conference on Connected Health: Applications, Systems and Engineering Technologies (CHASE).

[105] Bagavathyraj H., Joseph R., Madathil S.C. (2024). Vehicle Detection, Classification, and Re-Identification Using AI: A Systematic Review. IEEE Access.

[106] Hofstra G., Roelofs J., Rutter S.M., van Erp-van der Kooij E., de Vlieg J. (2022). Mapping Welfare: Location Determining Techniques and Their Potential for Managing Cattle Welfare—A Review. Dairy.

[107] Al-Alawneh J., Rablee A.R., Olchowy T., McGowan M., Stevenson M.A., Clay S., McCready T. (2015). Review of Diagnostic Technologies for Monitoring Feedlot Animal Health.

[108] Sadrzadeh Ahmadi N. (2024). Automated Monitoring of Grooming Behaviour in Dairy Cows. Master’s Thesis.

[109] Jongeneel R., Brouwer F., Farmer M., Müssner R., Roest K.D., Poux X., Fox G., Meister A., Karaczun Z., Winsten J. (2007). Compliance with Mandatory Standards in Agriculture: A Comparative Approach of the EU Vis-à-vis the United States, Canada and New Zealand.

[110] Hocquette J.F., Botreau R., Legr I., Polkinghorne R., Pethick D.W., Lherm M., Picard B., Doreau M., Terlouw E.M. (2014). Win–win strategies for high beef quality, consumer satisfaction, and farm efficiency, low environmental impacts and improved animal welfare. Anim. Prod. Sci..

[111] Edwards-Callaway L., Davis M., Dean L., McBride B. (2024). Stakeholder perceptions of animal welfare as a component of sustainable beef programs in the United States—A pilot study. Animals.

[112] Estrella M.B., Renee Ching M.D. (2024). Development of an Adoption Framework for Precision Livestock Farming (PLF): A Case in a Developing Economy. Lecture Notes in Electrical Engineering.

[113] Behrendt K., Takahashi T., Rutter M. Precision Livestock Farming technologies—At what cost? An ex ante analysis of technologies and digitalisation in grazing systems. Proceedings of the INFER Workshop on Agri-Tech Economics.

[114] Caja G., Castro-Costa A., Salama A.A., Oliver J., Baratta M., Ferrer C., Knight C.H. (2020). Sensing solutions for improving the performance, health and wellbeing of small ruminants. J. Dairy Res..

[115] O’Shea R., O’Donoghue C., Ryan M., Breen J. Understanding farmers: From adoption to attitudes. Proceedings of the European Association of Agricultural Economists, 166th Seminar.

[116] Zhang Z., Wilson C.-A., Hay R., Everingham Y., Naseem U. (2025). BeefBot: Harnessing Advanced LLM and RAG Techniques for Providing Scientific and Technology Solutions to Beef Producers. Proceedings of the 31st International Conference on Computational Linguistics: System Demonstrations.

[117] Ferreira R.E.P., Dórea J.R.R. (2025). International Symposium on Ruminant Physiology: Leveraging computer vision, large language models, and multimodal machine learning for optimal decision making in dairy farming. J. Dairy Sci..

